# Effect of Mutations in GvpJ and GvpM on Gas Vesicle Formation of *Halobacterium salinarum*

**DOI:** 10.3389/fmicb.2021.794240

**Published:** 2021-12-16

**Authors:** Alisa Jost, Regine Knitsch, Kerstin Völkner, Felicitas Pfeifer

**Affiliations:** Microbiology and Archaea, Department of Biology, Technical University Darmstadt, Darmstadt, Germany

**Keywords:** protein–protein interaction, split-GFP, *Haloferax volcanii*, substitution variants, accessory Gvp proteins

## Abstract

The two haloarchaeal proteins, GvpM and GvpJ, are homologous to GvpA, the major gas vesicle structural protein. All three are hydrophobic and essential for gas vesicle formation. The effect of mutations in GvpJ and GvpM was studied in *Haloferax volcanii* transformants by complementing the respective mutated gene with the remaining *gvp* genes and inspecting the cells for the presence of gas vesicles (Vac^+^). In case of GvpJ, 56 of 66 substitutions analyzed yielded Vac^–^ ΔJ + J_mut_ transformants, indicating that GvpJ is very sensitive to alterations, whereas ten of the 38 GvpM variants resulted in Vac^–^ ΔM + M_mut_ transformants. The variants were also tested by split-GFP for their ability to interact with their partner protein GvpL. Some of the alterations leading to a Vac^–^ phenotype affected the J/L or M/L interaction. Also, the interactions J/A and J/M were studied using fragments to exclude an unspecific aggregation of these hydrophobic proteins. Both fragments of GvpJ interacted with the M1–25 and M60–84 fragments of GvpM, and fragment J1–56 of GvpJ interacted with the N-terminal fragment A1–22 of GvpA. A comparison of the results on the three homologous proteins indicates that despite their relatedness, GvpA, GvpJ, and GvpM have unique features and cannot substitute each other.

## Introduction

Gas vesicles are proteinaceous, gas-filled structures synthesized by several bacteria and archaea. They are spindle- or cylinder-shaped structures with conical end caps, and their protein wall is mainly constituted by the gas vesicle protein GvpA (Walsby, 1994; Pfeifer, 2012). This 8-kDa hydrophobic protein aggregates into 4.6-nm-wide ribs running as helix perpendicular to the long axis (Offner et al., 1998). The 42-kDa GvpC is attached to the exterior surface to stabilize the wall that lacks any lipid. Both proteins are encoded by the *gvpACNO* transcription unit in the haloarchaeon *Halobacterium (Hbt.) salinarum.* Eight additional Gvp proteins are involved in gas vesicle formation, encoded by the transcription unit *gvpFGHIJKLM* located upstream of *gvpA* in opposite orientation (Englert et al., 1992; DasSarma, 1993). Both clusters are separated by the *gvpDE* genes encoding the two endogenous regulatory proteins (Hofacker et al., 2004; Scheuch and Pfeifer, 2007; Marschaus and Pfeifer, 2012). All three transcription units are clustered in the so-called p-vac region on plasmid pHH1. The function of the *gvp* genes has been investigated by transformation experiments using the related haloarchaeon *Haloferax volcanii* as host; this strain is easier to transform, grows faster, and lacks any of the *gvp* genes. *Hbt. salinarum* strain PHH1 contains the constitutively expressed p-vac region and, in addition, the related c-vac region expressed in the stationary growth phase only (Englert et al., 1992). The related *Hbt. salinarum* strain NRC-1 contains two copies of the *gvp1* gene cluster (almost identical to p-vac) and the *gvp2* gene cluster (closely related to c-vac) on two mini-chromosomes (Ng et al., 2000). Except for GvpK and the two regulatory proteins GvpD and GvpE, all Gvp proteins have been identified in gas vesicle preparations (Shukla and DasSarma, 2004; Chu et al., 2011).

The two accessory proteins GvpJ (12.6 kDa) and GvpM (9.2 kDa) derived from the p-vac region are the subject of this paper, and both exhibit sequence similarities to GvpA (A-M, 48%; A-J, 50%; J-M, 60%) (Supplementary Figure 1). The three homologous proteins are essential, since a deletion of any of these genes results in gas vesicle negative (Vac^–^) transformants (Offner et al., 2000). They are grouped in the A-J-M family of hydrophobic gas vesicle proteins. An *in silico* structural model of GvpA is available and predicts a coil–α–β–β–α–coil structure (Strunk et al., 2011; Ezzeldin et al., 2012), also supported by solid state NMR analysis (Sivertsen et al., 2010; Daviso et al., 2013). Using His-tagged Gvp for pull-down experiments on a Ni-NTA matrix shows that GvpM interacts with GvpH, GvpJ, and GvpL (Tavlaridou et al., 2014). More recent experiments with fragments of GvpM confirm the M/H and M/L interaction with the split-GFP method, but the M/J interaction was not detectable, presumably due to the formation of unspecific aggregates that negatively affect the assembly of GFP (Winter et al., 2018). However, tagging GvpM with the cellulose-binding domain CBD demonstrates that _CBD_M selects all other accessory Gvp (F, G, H, I, J, K, and L) (Völkner et al., 2020). Since all of them interact with other Gvp proteins and were also selected by _CBD_M in _CBD_M + FGHIJKL transformants, we speculate that they form (a) complex(es) during gas vesicle formation. Since the *gvpFGHIJKLM* co-transcript occurs in early exponential growth only, these proteins are required in early stages of gas vesicle assembly (Offner and Pfeifer, 1995). In the case of the major gas vesicle protein GvpA, split-GFP analyses yield GvpF as the only interaction partner, and the interaction has been confined to the N-terminal A1–22 fragment of GvpA containing the first 22 aa including α-helix 1 (α1) (Völkner et al., 2020).

A scanning mutagenesis performed with GvpA points out important amino acid (aa) residues required to form a functional gas vesicle (Strunk et al., 2011; Knitsch et al., 2017). The single aa substitutions in the 76-aa GvpA were analyzed in *Hfx. volcanii* ΔA + A_mut_ transformants. Construct ΔA contains all *gvp* genes of p-vac in pWL102 except for *gvpA*, and A_mut_, the mutated *gvpA* inserted in pJAS35. Both shuttle vectors are low in copy number and compatible, allowing the complementation. While ΔA + A_wt_ transformants (A_wt_ expresses *gvpA* wild type) are Vac^+^ and form gas vesicles similar to *Hbt. salinarum* PHH1, different Vac phenotypes occur with ΔA + A_mut_ transformants. Approximately 43% are Vac^–^, and most of the Vac^+^ transformants contain spindle-shaped gas vesicles as the wild type, but some harbor long, cylinder-shaped gas vesicles, and a few form mini gas vesicles (Knitsch et al., 2017). In addition to the alterations in shape, the amount of gas vesicles could vary from a few to up to 60–70 per cell as found with ΔA + A_wt_ transformants. Most mutations leading to a Vac^–^ phenotype concern aa residues pointing to the outside of the protein structure implying that they contact other GvpA or accessory Gvp. Many of these are found in α-helix 1, β-sheet 2, and loop 3, whereas the second half of α2 turned out to be not important for gas vesicle formation (Knitsch et al., 2017).

A similar but not as detailed mutational analysis has been performed with the 83-aa GvpM; twenty aa were substituted in the α–β–β–α portion related to GvpA, and small deletions were introduced at the N- or C-terminus (Tavlaridou et al., 2014; Winter et al., 2018). Six of these substitution variants yield Vac^–^ ΔM + M_mut_ transformants. The gas vesicles found in Vac^+^ transformants are all of wild-type shape, and ten of these produce gas vesicles in similar amounts as wild type, but four contain a few gas vesicles only (Vac^±^). A deletion of 5 aa at the N-terminus of GvpM (M_Δ5N_) leads to a Vac^±^ phenotype, whereas ΔM + M_Δ10N_ transformants are Vac^–^ (Tavlaridou et al., 2014). At the C-terminus, ΔM + M_Δ25C_ transformants are Vac^±^, but transformants carrying M_Δ27C_ are Vac^–^, suggesting that the last 25 aa of GvpM are not required to form a functional gas vesicle (Winter et al., 2018). Interaction studies by split-GFP performed with fragments of GvpM identified the N-terminal 25 aa as important for the interaction with GvpL (relative fluorescence, rf 45), and the C-terminal 25 aa contact GvpF, GvpH, and GvpL (rf 12 in each case). This implies that there are two sites in GvpL interacting with GvpM (Winter et al., 2018).

In this report, we investigated additional substitutions especially in the N-terminal 25-aa fragment of GvpM to determine the interaction site with GvpL more precisely. The substitutions in GvpM were also analyzed in ΔM + M_mut_ transformants for their ability to form gas vesicles. The Vac^–^ phenotype due to a GvpM variant might be caused at least in part by an altered L/M interaction. Also, the related GvpJ protein was examined by single aa substitutions within the α–β–β–α portion, and by deletions at the N- or C-terminus, and each of these GvpJ variants was tested in ΔJ + J_mut_ transformants for the ability to support gas vesicle formation. Many of these variants yielded Vac^–^ transformants, indicating that GvpJ is very sensitive to alterations. We performed interaction studies with GvpJ_mut_ and GvpL to determine aa affecting the J/L interaction. Finally, the interaction of GvpA, GvpM, and GvpJ was studied by split-GFP using fragments of these proteins to exclude an unspecific aggregation *via* the hydrophobic central portions. An interaction was observed between GvpJ and GvpM as well as between GvpJ and GvpA. Additional interaction partners of GvpJ were identified by investigating the other accessory proteins for interactions with the hydrophilic C-terminal half of GvpJ.

## Materials and Methods

### Strains and Cultivation Conditions

*Escherichia coli* strains Top10F (Invitrogen by Life Technologies) and GM1674 (*dam*^–^) (Palmer and Marinus, 1994) were incubated in Luria-Bertani medium supplemented with ampicillin (100 μg/ml) under shaking at 37°C overnight. Incubation on solid media was performed overnight in LB media containing a similar ampicillin concentration. *Hfx. volcanii* strain WFD11 (plasmid pHV2 deleted; Cline et al., 1989) or WR340 (*his* mutation; Bitan-Banin et al., 2003) was incubated in 3 M VM medium [3 M NaCl, 150 mM MgSO_4_, 50 mM KCl, 0.05% (w/v) CaCl_2_, 25 mM Tris–HCl, pH 7.2, 10 nM MnCl_2_, 0.5% (w/v) tryptone, 0.3% (w/v) yeast extract], and histidine [0.02% (w/v)] was added for WR340 in addition. Transformation was done according to Pfeifer et al. (1994), and transformants were selected by the addition of 6 μg/ml mevinolin (for selection of pWL102 or pWL_fdx_) and/or 0.2% novobiocin (for selection of pJAS35 or pMDS20). For solid media, 1.8% agar was added and plates were incubated for 5–7 days at 42°C in a ziplock bag including a wet paper towel to prevent dehydration and the formation of salt crystals. When colonies were examined for the Vac phenotype, the plates were incubated for up to 5 weeks in the dark at room temperature. Liquid cultures were incubated at 42°C under shaking for 3–4 days. Cultures used to quantify the fluorescence by split-GFP were incubated for 24 h at 37°C, followed by 24 h at 30°C, always shaking at 180 rpm (Winter et al., 2018).

### Vector Constructions and Mutagenesis of *gvp* Genes

The ΔJ construct contains except for *gvpJ* all genes of the p-vac region inserted in vector pWL102 (Lam and Doolittle, 1989). The vector construction was achieved by Gibson assembly (Gibson et al., 2009; Gibson, 2011) using NEBuilder^®^ HiFi DNA Assembly Master Mix. The two fragments M-K and I-O of p-vac were amplified by PCR using *Hbt. salinarum* PHH1 DNA as template and inserted in pWL102 using the *Nco*I site. Complementation of the construct in *Hfx. volcanii* WFD11 transformants was performed with *gvpJ* (wild type or variant) inserted in the expression vector pJAS35. The mutagenesis of the *gvpJ* reading frame was performed in *E. coli* inserted in pBSKII+. The nucleotides encoding the desired substitution were altered by site-directed mutagenesis PCR, and oligonucleotides containing the desired alteration are presented in Supplementary Table 1. The mutated *gvpJ* reading frame was transferred to pJAS35 as a *Pst*I-*gvpJ*-*Acc*65I fragment. A similar procedure was applied to obtain the mutations in *gvpM*. The ΔM construct (p-vac region lacking *gvpM*, inserted in pWL102; Offner et al., 2000) and *gvpM* inserted in pJAS35 (Tavlaridou et al., 2014) were used to transform *Hfx. volcanii* WR340. The *gvpM* gene in pJAS35 served as a template for site-directed mutagenesis PCR. The mutation was introduced *via* oligonucleotides used as primers for amplification by PCR (Supplementary Table 1). In each case, the presence of the desired mutation was verified by DNA sequence analysis.

For the split-GFP analysis, the previously described vectors pJAS-NGFP-Nterm (_N_X – NGFP fused at the N-terminus of GvpX) and -Cterm (X_N_ – NGFP fused at the C-terminus), as well as pWL_fdx_-CGFP-Nterm (_C_X) and -Cterm (X_C_) were used (Winter et al., 2018). These vectors either contain the N-terminal or C-terminal portion of the *mgfp2* reading frame encoding the salt-stable mGFP2 (Born and Pfeifer, 2019). The split in mGFP2 occurs between aa 157 and 158 resulting in the N-terminal (NGFP) and C-terminal (CGFP) portions, respectively. The fusion of *mgfp2* fragments to *gvp* includes a 14-aa (pJAS) or 16-aa (pWL_fdx_) linker region. In both vectors, the reading frames are expressed under the control of the constitutive *P*_fdx_ promoter. The vectors containing the *gvpA*, *gvpJ*, and *gvpM* of p-vac, or the fragments A1–22, A44–76, M(25N), and M(25C), have been described previously (Winter et al., 2018; Völkner et al., 2020). The *gvp* sequences encoding the proteins or peptides used for the protein–protein interaction studies in this report were amplified by PCR using the p-vac region as template. The oligonucleotides used are listed in Supplementary Table 1. For the insertion of fragments in pJAS derivatives, the *Nco*I and *Blp*I restriction sites were introduced; for pWL_fdx_-Nterm, the *Bam*HI and *Kpn*I restriction sites; and for pWL_fdx_-Cterm, the *Nco*I and *Bam*HI restriction sites. In some cases, *Bsp*HI was used instead of *Nco*I, taking advantage of the ligation of compatible ends. To insert the *gvpJ*_*mut*_ reading frames into the split-GFP vector pWL-CGFP-Cterm, the existing substitutions in *p-gvpJ* × pJAS35 were used. The fragments were inserted as *Nco*I-*gvpJ*_*mut*_-*Bam*HI amplicons (pJ_mutC_). The same procedure was performed with the *gvpM*_*mut*_ reading frames, and the existing mutation plasmids (Tavlaridou et al., 2014) were applied. For the substitutions in M1–25, plasmid pM(25N) × pJAS35-NGFP-Cterm [pM(25N)_N_] was used as a template for site-directed mutagenesis PCR.

### Western Blot Analysis

To analyze whether the GvpJ and GvpM variants were indeed produced, Western analysis was performed. For this purpose, 50 ml of 3M VM (His) media with the appropriate antibiotic supplementation were inoculated with the respective *Hfx. volcanii* transformants and cultured at 42°C. The cells were harvested in the early stationary growth phase by centrifugation at 2,370 × *g*, 30 min, 4°C. The cells were resuspended in lysis buffer, DNase I (0.1 mg/ml) was added, and the solution was incubated for 3 h at 37°C, followed by dialysis against Tris–HCl, pH 7.2, overnight. The cell extract was centrifuged to remove cell debris, and the protein concentration was determined by Bradford assay. Twenty micrograms of protein was separated by SDS-PAGE (Schägger and von Jagow, 1987) and transferred to a PVDF membrane (Roti Fluoro PVDF, Carl Roth). The membrane was dried, reactivated with 100% (v/v) methanol, and washed 2 times with PBS (137 mM NaCl, 2.7 mM KCl, 10 mM Na_2_HPO_4_, and 2 mM KH_2_PO_4_) before blocking with Odyssey Blocking Buffer (Licor) for 1–2 h. The membrane was incubated overnight with the respective antiserum raised against GvpA, GvpJ, or GvpM and then washed 4 times for 5 min with PBS + 0.1% Tween20 (v/v). The membrane was incubated for 2–3 h with the secondary antibody IRDye 800 CW (Licor) coupled to a fluorophore, and washed 4 times for 5 min with PBS + 0.1% Tween20. Excess Tween20 was removed by washing with PBS. Detection of the secondary antibody was performed at 800 nm using Odyssey Fc Imager (Licor).

### Quantitation of GFP Fluorescence

To measure the fluorescence, cultures were grown as described above. After 48 h (OD_600_ 1.5–2), 2 ml of the culture was harvested (2 min at 9,600 × *g*, 20°C), and the cell in the sediment was washed with 1 ml of basal salts (3 M NaCl, 150 mM MgSO_4_, and 50 mM KCl) and resuspended in 500 μl of basal salts. The cell concentration was adjusted to OD_600_ 1, and 300 μl was transferred to a microtiter plate. Two biological replicates each with three technical replicates were investigated. The fluorescence was measured with a phosphor imager in LAU/mm^2^. Using the intrinsic fluorescence of the cells (untransformed *Hfx. volcanii*), the relative fluorescence was calculated by subtracting the fluorescence of *Hfx. volcanii* WR340 and dividing the value obtained with the fluorescence of WR340 (Winter et al., 2018). The original data of the experiments described in this report are presented in Supplementary Tables 3–5.

### Isolation of Gas Vesicles and Transmission Electron Microscopy

Gas vesicles were isolated from transformants grown in colonies on solid media for 5 weeks. A few of the colonies were taken and transferred to 1 ml of 1 mM MgSO_4_ containing 10 μg/ml DNase I. The cells were lysed for 3 h on an overhead rotator to release the gas vesicles. The mixture was centrifuged for 2 h at 95 × *g* and 4°C. Gas vesicles float to the surface and settle as a white layer at the edge of the vessel. They were removed with a pipette and transferred to 500 ml of 100 mM Tris–HCl, pH 7.2, plus 5% NaCl. The gas vesicle suspension was used for transmission electron microscopy and can be stored in the refrigerator for months.

Cells of the transformants or isolated gas vesicles were inspected by TEM. Twenty microliters of gas vesicle suspension, or cells resuspended in 20 μl of basal salts, were transferred to a formvar-coated copper grid (300 mesh, Plano GmbH). The suspension was incubated on the grid for 1 min, and the liquid was removed with a Whatman 3 M paper. Images were taken using a Zeiss EM109 microscope and Gatan Multiscan 600 W camera.

## Results

### Effect of Mutations in GvpJ on Gas Vesicle Formation

The 114-aa GvpJ protein is the largest protein of the A-J-M family, and the amino acid sequence is 60% similar to GvpM and 50% similar to GvpA (Supplementary Figure 1). The conserved sequences are confined to the N-terminal half and include the α–β–β–α structure predicted by *in silico* modeling as well as two sequence motifs, 46-RAAIA-50 and 57-EYGL-60, that are present in GvpJ and GvpM only. The C-terminal portion of GvpJ (aa 62–114) is unique and hydrophilic. Only three aromatic amino acids (F52, Y58, and F62) are present in GvpJ, and cysteine is lacking. The *in silico* 3D structure of GvpA has been used to calculate a homology structure of GvpJ using I-Tasser server (Winter et al., 2018).

**FIGURE 1:**
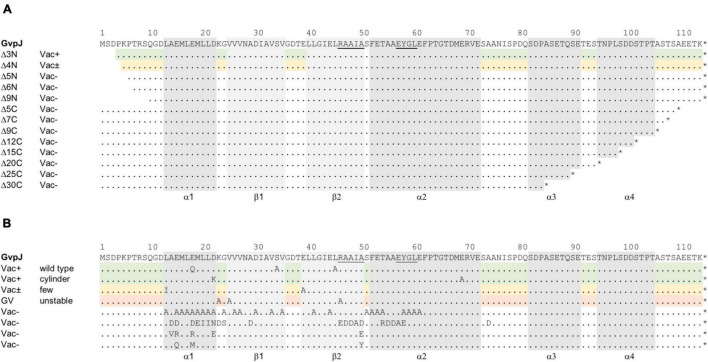
Mutations in the accessory protein GvpJ. The aa sequence of GvpJ is given on top with the α-helices shaded in gray; the β-sheets are shaded in light gray. These structural features are marked below. Dots indicate identical aa residues. The RAAIA and EYGL motifs are underlined. **(A)** Deletions introduced in GvpJ. Each line represents a deletion variant, and the Vac phenotype is given on the left. Green shades highlight Vac^+^ and yellow shades Vac^±^ transformants. **(B)** Summary of the single aa substitutions leading to a Vac^+^, Vac^±^, or Vac^–^ phenotype. Each letter represents a single aa substitution in GvpJ. Different substitutions are arranged according to the Vac phenotype. The Vac^+^ phenotype is divided in gas vesicle of wild-type or cylinder shape (shaded in green). The line containing single substitutions in GvpJ yielding Vac^±^ ΔJ + J_mut_ transformants is shaded in yellow, and the line with alterations yielding unstable gas vesicles is shaded in red.

Single aa substitutions (mainly by alanine; other aa were used for the substitution when alanine was present in the original sequence) and successive deletions at the N- or C-terminus were introduced to analyze the effect of these alterations on gas vesicle formation in *Hfx. volcanii* ΔJ + J_mut_ transformants (Figure 1). Construct ΔJ was produced using the Gibson Assembly (Gibson et al., 2009; Gibson, 2011) as described in *Materials and Methods*; the construct contains all *gvp* genes of the p-vac region inserted in shuttle vector pWL102 except for *gvpJ*. The *gvpJ* reading frame (wild type or variant) was inserted in vector pJAS35 and expressed under *P*_*fdx*_ promoter control (construct J_wt_ or J_mut_). The colonies of each ΔJ + J transformant were inspected for their Vac phenotype after 5 weeks, and transmission electron microscopy (TEM) was performed to confirm the presence or absence of gas vesicles. Vac^+^ colonies are turbid and pink in color, whereas Vac^–^ colonies are transparent and red. The colonies of the ΔJ + pJAS35 (empty vector) transformants were Vac^–^, whereas ΔJ + J_wt_ transformants formed turbid colonies that were filled with gas vesicles (Supplementary Figure 2). TEM analyses supported the presence of gas vesicles. Thus, the effect of the altered GvpJ on gas vesicle formation could be studied in the respective ΔJ + J_mut_ transformants.

The deletions introduced in GvpJ encompassed up to 9 aa at the N-terminus (variants J_Δ3N_ through J_Δ9N_), or between 5 and 30 aa at the C-terminus (J_Δ5C_ through J_Δ30C_) (Figure 1A). The phenotypes of the resulting transformants are shown in Supplementary Figure 2. Colonies of ΔJ+J_Δ3N_ transformants were Vac^+^, whereas ΔJ+J_Δ4N_ transformants were Vac^±^ and contained a few gas vesicles only. The ΔJ + J_Δ5N_, ΔJ + J_Δ6 N_ and ΔJ + J_Δ9N_ transformants were Vac^–^, suggesting that essential aa were deleted. Thus, only 4 aa of the N-terminus could be deleted without affecting gas vesicle production. All deletions at the C-terminus of GvpJ (J_Δ5C_ through J_Δ30C_) yielded Vac^–^ transformants. To ensure that the Vac^–^ phenotype was not due to a lack of GvpJ, Western analyses were performed. Except for J_Δ3N_, all GvpJ deletion variants were detectable in ΔJ + J_mut_ transformants (Supplementary Figure 3A). However, the ΔJ + J_Δ3N_ transformants were Vac^+^ and thus produced GvpJ_Δ3N_ (Supplementary Figure 2). These results demonstrated that a deletion of five aa at the N-terminus, or of five aa at the C-terminus of GvpJ already prevented the gas vesicle formation implying that almost all of the GvpJ sequence is essential.

**FIGURE 2:**
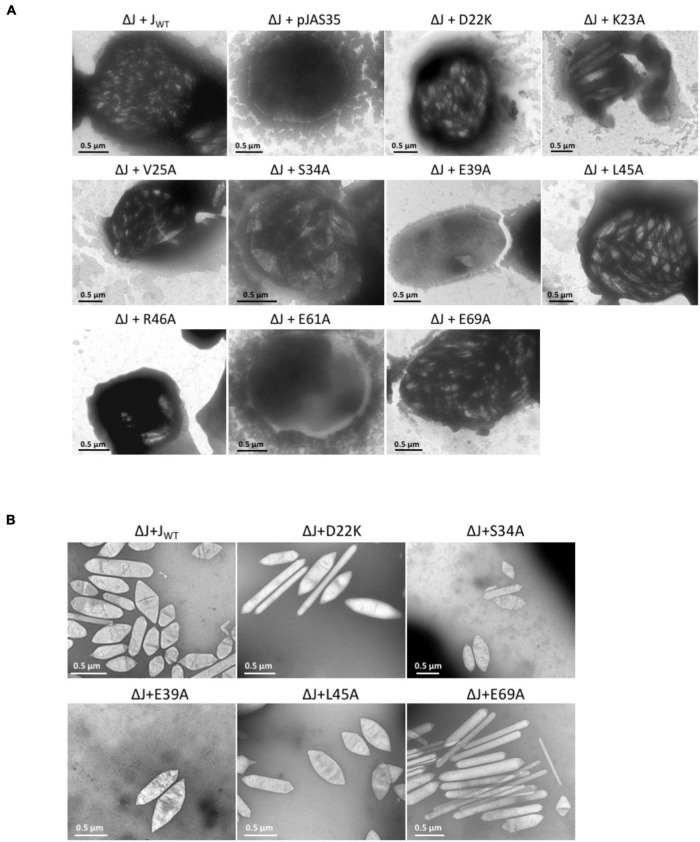
Electron micrographs of *Hfx. volcanii* ΔJ + J_wt_ or ΔJ + J_mut_ transformants, and of isolated gas vesicles. The respective substitutions in GvpJ of the ΔJ + J_mut_ transformants are indicated on top. **(A)** Representative cells of the different Vac^+^ or Vac^–^ transformants. Gas vesicles are seen as particles inside the cells. **(B)** Isolated gas vesicles of ΔJ + J_wt_ and ΔJ + J_mut_ transformants.

The aa substitutions performed with GvpJ included an alanine scan in the conserved 60-aa N-terminal sequence encompassing most of the α–β–β–α structure. In total, 34 single aa were substituted by alanine, and an additional 30 aa were substituted by another aa, especially when an alanine residue was present in the original GvpJ sequence (Figure 1B). Western analyses performed with each of the ΔJ + J_mut_ transformants confirmed that the GvpJ variants were all produced (see Supplementary Figure 3). GvpJ was detected as a monomer, but also as a dimer that was not seen in the lysate of the positive control J_WT_. Only ten of these ΔJ + J_mut_ transformants contained gas vesicles (see Figure 2A and Supplementary Table 2). Gas vesicles were isolated and investigated by TEM. The analysis yielded cylinder-shaped gas vesicles in the case of JE_69A_ and J_D22K_; the latter variant led to a mixture of cylinder- and spindle-shaped structures (Figure 2B). The other transformants contained gas vesicles of wild-type shape. The ΔJ + J_L13I_ and ΔJ + J_E39A_ transformants produced only a few gas vesicles per cell and were Vac^±^ (see Figure 2A). However, the gas vesicles observed in ΔJ + J_mut_ transformants containing the GvpJ variants K23A, V25A, and R46A could not be isolated. The isolation involves a flotation step, and the gas vesicles produced with these variants were unable to float. These alterations in GvpJ appear to affect the gas vesicle stability. An overview of all these results in respect to the GvpJ sequence and the Vac phenotype is presented in Figure 1B. The substitutions in GvpJ leading to Vac^–^ ΔJ + J_mut_ transformants cluster in α-helix 1, but also the aa of the β-sheet region turned out to be important. A Vac^–^ phenotype was also obtained with alterations in the RAAIA and EYGL motifs that are present in GvpJ and GvpM, underlining that these conserved sequences are important for the function of GvpJ in gas vesicle assembly. Overall, an exceptionally high percentage of 84% of the ΔJ + J_mut_ transformants was Vac^–^ and unable to form gas vesicles.

### Interaction Studies of GvpJ_mut_ and GvpL

**FIGURE 3:**
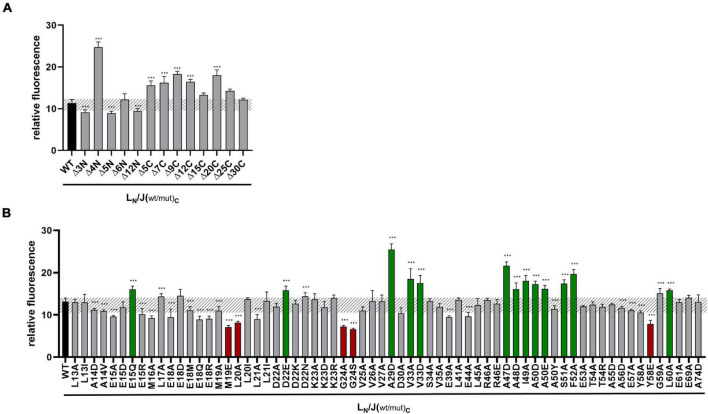
Split-GFP analyses of GvpL and variants of GvpJ. **(A)** Rf values of GvpL and deletion variants of GvpJ. The wild type version L_C_/J_N_ is labeled in black. L_C_, CGFP is fused to the C-terminus of GvpL; J_N_, NGFP to the C-terminus of GvpJ. **(B)** Rf values of L/J_wt_ (black) and the various L/J_mut_ *Hfx. volcanii* transformants with single substitutions in GvpJ. The respective substitution is marked on the bottom. Transformants showing a significantly lower fluorescence are labeled in red, and transformants with a higher fluorescence are in green. The significance of the rf values was calculated by Student’s *t*-test against wild type. ^***^extremely significant = *p* ≤ 0.001.

Previous analyses indicated that GvpJ is able to interact with the accessory protein GvpL when analyzed by CBD-tagged GvpL, or by split-GFP (Völkner et al., 2020). A relative fluorescence of rf 11.3 was determined for the L/J interaction. The effect of the mutations in GvpJ on the interaction with GvpL was studied by split-GFP in the combination J_C_/_N_L, which yields the highest fluorescence of transformants (Völkner et al., 2020). The GvpJ variants were fused with the CGFP fragment at the C-terminus, whereas NGFP was fused at the N-terminus of GvpL. If GvpJ and GvpL interact, a fluorescent GFP is formed; the fluorescence of the respective transformant was determined and the relative fluorescence (rf value) was calculated (Supplementary Table 3). In addition, the respective J_(mut)C/N_L transformants were analyzed by Western analysis for the presence of GvpL and GvpJ. The transformants contained both proteins (Supplementary Figure 4). The wild-type combination J_C_/_N_L yielded a relative fluorescence of rf 12–16, and the transformants with GvpJ carrying a deletion at the C-terminus (Δ5C through Δ30C) indicated a similar fluorescence, implying that the last 30 aa GvpJ are not required for the J/L interaction (Figure 3A). The deletions at the N-terminus yielded a high fluorescence in the case of the J_Δ4NC/N_L transformants, whereas the fluorescence of the transformants containing J_Δ5NC_ or J_Δ6NC_ was similar to the wild type (Figure 3A).

**FIGURE 4:**
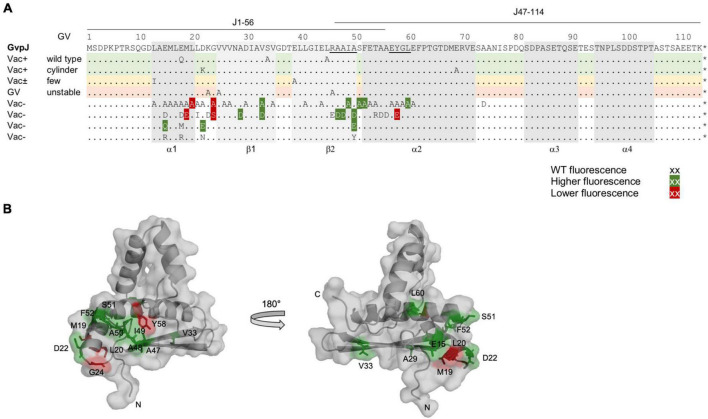
Summary of the fluorescence of L/J_mut_ transformants in relation to the Vac phenotype of the respective ΔJ + J_mut_ transformants. **(A)** Amino acid sequence of GvpJ; the α-helices and β-sheets are shaded in gray. These structural features are marked at the bottom of the alignment. The RAAIA and EYGL motifs are underlined. The lines on top indicate the aa sequence present in fragments J1–56 and J47–116. A summary of the single aa substitutions resulting in a Vac^+^, Vac^±^, or Vac^–^ phenotype is given below. Dots represent aa identical to GvpJ, and each letter represents a single aa substitution in J_mut_. Substitutions yielding a significantly higher fluorescence in L/J_mut_ transformants are shaded in dark green, whereas a lower fluorescence is shaded in red. **(B)** Homology structural model of GvpJ and location of the aa leading to a higher (green) or a lower (red) fluorescence. The homology model of GvpJ is based on the *in silico* model of GvpA (Strunk et al., 2011) and was calculated by the I-Tasser server (Zhang, 2008; Roy et al., 2010; Yang et al., 2015).

In case of the single aa substitutions, a lower fluorescence was observed with the five GvpJ variants M19E, L20A, G24A/-S, and Y58E, and a significantly higher fluorescence was obtained with the variants E15Q, D22E, A29D, V33A/-D, A47D, A48D, I49A, A50D/-E, S51A, F52A, and L60A (Figure 3B). The substitutions concerning pos. 47–50 in GvpJ affected the RAAIA motif, and Y58 and L60 are part of the EYGL motif (Figure 4A). The results suggested that RAAIA/EYGL are involved in the J/L interaction. Variants M19A, L20A, and G24A cluster in α1 and loop 1, and a substitution at one of these positions negatively affected the J/L interaction. All other GvpJ variants yielded a fluorescence similar to the wild-type J_C_/_N_L transformants. The aa substitutions leading to a higher or a lower fluorescence are marked in the structural model of GvpJ (Figure 4B). It is possible that GvpL interacts with GvpJ in this region. A comparison of these results to the Vac phenotype of the respective ΔJ + J_mut_ transformants showed that all of these variants that resulted in an altered fluorescence yielded Vac^–^ ΔJ + J_mut_ transformants (Figure 4A).

### Effect of Mutations in GvpM on Gas Vesicle Formation

**FIGURE 5:**
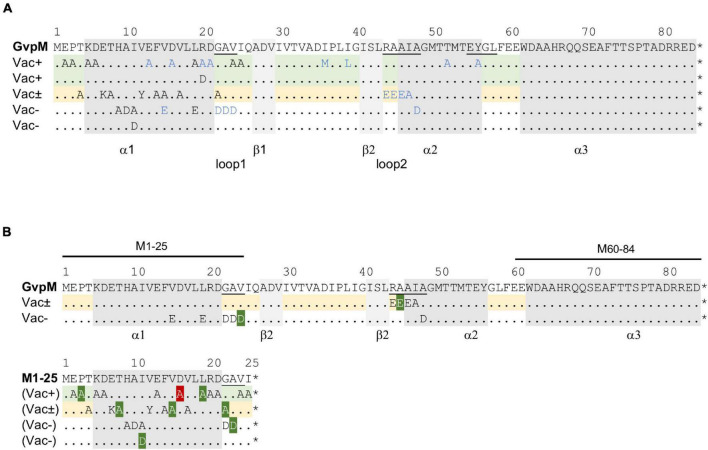
Mutations in GvpM and in fragment M1–25. **(A)** Amino acid sequence of GvpM with the α-helices (gray) and β-sheets (light gray). Dots indicate aa identical to GvpM. The GAV, RAAIA, and EYGL motifs are underlined. **(A)** Summary of the single aa substitutions leading to a Vac^+^, Vac^±^ or Vac^–^ phenotype. Each letter represents a single aa substitution. The blue letters are substitutions investigated by Tavlaridou et al. (2014). The lines indicating substitutions in GvpM yielding Vac^+^ ΔM + M_mut_ transformants are shaded in green, and the lines with substitutions yielding a Vac^±^ phenotype are in yellow. **(B)** Summary of the fluorescence of L/M_mut_ or L/M1–25_mut_ transformants in relation to the Vac phenotype of the respective ΔM + M_mut_ transformants. The lines on top of GvpM indicate the aa present in fragments M1–25 and M60–64. The aa substitutions yielding a higher fluorescence are shaded in dark green, whereas the D16A substitution yielding a significantly lower fluorescence of the respective L/M1–25_D16A_ transformant is shaded in red. *marks the end of the respective sequence.

Previous analyses of GvpM indicated that the N-terminal 25-aa fragment plays an important role in the interaction with GvpL and in the formation of gas vesicles (Winter et al., 2018). To define the putative M/L interaction site in GvpM in further detail, additional substitutions were introduced in the N-terminal region of GvpM and the resulting proteins investigated in ΔM + M_mut_ transformants for their ability to form gas vesicles. The ΔM construct contains, except for *gvpM*, all *gvp* genes of the p-vac region in pWL102 and M_mut_, the mutated *gvpM* reading frame in vector pJAS35 (Tavlaridou et al., 2014). The single substitutions introduced in GvpM are summarized in Figure 5A, which also includes the substitutions investigated in earlier studies (blue letters; Tavlaridou et al., 2014; Winter et al., 2018). The colonies of the transformants were inspected for their Vac phenotype, and the cells were analyzed by TEM (Supplementary Figure 5). Western analyses confirmed that the GvpM protein was present in all transformants (Supplementary Figure 6). The 12-kDa GvpM monomer was detected in all cases, indicating that the Vac^–^ phenotype observed for some of these transformants was not due to the lack of GvpM.

Twenty substitutions in the N-terminal 25 aa of GvpM were studied in ΔM + M_mut_ transformants (Table 1), and gas vesicles were observed with variants E02A, P03A, K05A, D06A, L19A, V24A, and I25A (Supplementary Figure 5A). The amount of gas vesicles per cell and also the gas vesicle shape was similar to the ΔM + M_wt_ transformants used as control. Colonies producing the GvpM variants T04A, E07K, T08A, V12Y, F14A, V15A, V17A, or G22A were slightly turbid and red (Vac^±^), and TEM analyses indicated that about half of the cells contained a few (1–5) gas vesicles only, whereas the other half was Vac^–^ (Supplementary Figure 5B). Most of the gas vesicles were of wild-type shape, but in the case of the variants V12Y and G22A, the gas vesicles were twice as long. Colonies of ΔM + M_mut_ transformants carrying the variants H09A, A10D, I11A, or I11D were red and transparent, and TEM confirmed that these cells were Vac^–^ (Supplementary Figure 5C). Thus, the His, Ala, and Ile residues (HAI) at pos. 9–11 are essential to obtain gas vesicles. These data supported earlier results on N-terminal deletions in GvpM (Winter et al., 2018); the Δ10N deletion (Vac^–^) includes H09 and A10 that yielded a Vac^–^ phenotype when substituted by alanine, and the Vac^±^ phenotype of the ΔM + M_Δ5N_ might be caused by the lack of threonine at position 04 (T04A), since all other aa substituted by alanine yielded Vac^+^ transformants. Overall, these analyses demonstrated that many aa at positions 4–19 and the GAV motif in loop 1 in GvpM were important for gas vesicle formation (Table 1). Earlier experiments already indicated that the RAAIA motif (pos. 44–48) was important, since substitutions resulted in Vac^±^ or Vac^–^ transformants (Tavlaridou et al., 2014).

**TABLE 1:** Effect of substitutions in GvpM on gas vesicle formation and on the M_mut_/L interaction.

Substitution	Structure In GvpM	Vac^#^ phenotype	Effect on M_mut_/L rf value#
E02A	N-term	wt	wt
P03A	N-term	wt	Higher
T04A	N-term	Few	wt
K05A	α1	wt	wt
D06A	α1	wt	wt
E07K	α1	Few	wt
T08A	α1	Few	Higher
H09A	α1	Negative	wt
A10D	α1	Negative	wt
I11A	α1	Negative	wt
I11D	α1	Negative	Higher
V12Y	α1	Few longer	wt
E13A*	α1	wt	wt
F14A	α1	Few	wt
V15A	α1	Few	Higher
V15E	α1	Negative	wt
D16A*	α1	wt	Lower
V17A	α1	Few	wt
L19E*	α1	Negative	wt
L19A	α1	wt	Higher
R20A*	α1	wt	wt
D21A*	α1	wt	wt
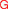 22A	Loop 1	Few, longer	Higher
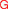 22D*	Loop 1	Negative	wt
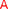 23D*	Loop 1	Negative	Higher
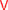 24D*	Loop 1	Negative	Higher
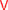 24A	Loop 1	wt	wt
I25A	Loop 1	wt	wt
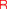 44E*	Loop 3	Few	wt
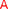 45E*	Loop 3	Few	Higher
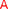 46E*	α2	Few	wt
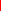 47A*	α2	Few	wt
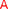 48D*	α2	Negative	wt

*GvpM variants constructed by Tavlaridou et al. (2014).

*^#^Vac phenotype: wt, similar to wild type; few = few gas vesicles.*

*Colors designate substitutions resulting in an altered Vac phenotype as well as an altered fluorescence (Vac^±^, green; Vac^–^, red).*

### Interaction Studies of GvpM_mut_ With GvpL

**FIGURE 6:**
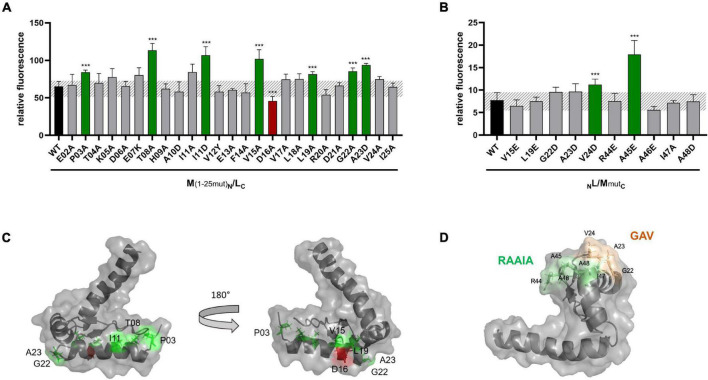
Split-GFP analyses of the interaction of GvpL with variants of GvpM or M1–25. **(A)** Rf values of L/M1–25 *Hfx. volcanii* transformants (black) and of various L/M1–25_mut_ transformants containing single substitutions in fragment M1–25. The respective substitution is indicated at the bottom. Transformants exhibiting a significantly lower fluorescence are labeled in red, and transformants showing a higher fluorescence are in green. **(B)** Rf values of GvpL and L/M wild type (black) and the various L/M_mut_ transformants. The significance was calculated by Student’s *t*-test. ^***^extremely significant = *p* ≤ 0.001. **(C)** Homology structure model of GvpM (Winter et al., 2018) showing the location of the aa where a substitution yielded a lower (red) or a higher (green) fluorescence of L/M or L/M1–25 transformants. **(D)** Location of the GAV and RAAIA motifs in GvpM.

To investigate a possible effect of these mutations on the interaction of GvpM with GvpL, split-GFP studies were performed. Previous analyses suggested that mainly the N-terminal 25-aa fragment of GvpM, M1–25 [=M (25N) in Winter et al. (2018)], interacts with GvpL (rf 45) (Winter et al., 2018). Many of the substitutions in this portion of GvpM also affected the gas vesicle formation in ΔM + M_mut_ transformants. Since the M1–25 fragment indicated a higher rf value in the L/M interaction studies than the entire GvpM, M1–25 was used as a template to introduce these substitutions. The M1–25_mut_ peptides were fused at the C-terminus to the N-terminal portion of GFP and tested in the M1–25_N_/L_C_ combination in *Hfx. volcanii* transformants for an interaction. The L_C_ construct produces GvpL fused at the C-terminus to the C-terminal portion of GFP (Winter et al., 2018). The control transformants M1–25_N_/L_C_ yielded the rf value 65, and similar values were determined for many of these M1–25_mut_ variants (Figure 6A). A significantly higher rf value was found with variants P03A, T08A, I11D, V15A, L19A, G22A, and A23D, and a lower rf value was only observed with variant D16A (Figure 6A). A comparison of these results to the Vac phenotype of the respective transformants is shown in Figure 5B and a summary of the data is given in Table 1. The aa altered in M1–25_mut_ yielding a higher relative fluorescence were mainly located on the surface of the structural model of GvpM and cluster in two regions (see Figure 6C).

The interaction with GvpL was also studied with substitutions in the entire GvpM protein and especially GvpM variants that incurred a substitution in the GAV- or RAAIA motif (pos. 44–48) (Figure 6B). The RAAIA motif is conserved between GvpM and GvpJ, and not present in GvpA that instead contains the related RVVAA sequence (Supplementary Figure 1). The substitutions in RAAI of GvpM result in a Vac^±^ phenotype, whereas ΔM + M_mut_ transformants with the alterations in GAV or A48D of RAAIA are Vac^–^ (Tavlaridou et al., 2014; Table 1). The GAV and RAAIA motifs are located in a similar region in the predicted structure of GvpM (Figure 6D). The fluorescence of the _N_L/M_mutC_ transformants containing the entire GvpM protein was much lower compared to the fluorescence found with M1–25 in _N_L/M1–25_C_ transformants (rf 8 vs. rf 65), indicating that a small peptide fused to a GFP-portion interacts and assembles GFP much easier (Figures 6A,B). The GvpM variants V24D (part of GAV) and A45E (last Ala of RAAIA) yielded a higher relative fluorescence than GvpM wild type, but all other variants yielded a similar rf value as found for _N_L/M_C_ transformants (Figure 6B). The variants V15A and L19A investigated with M1–25 resulted in a higher fluorescence than wild type, whereas the V15E and L19E substitutions in GvpM yielded a relative fluorescence similar to wild type (Figures 6A,B). Both aa residues point to the inside in the structural model of the protein and are presumably not contacting GvpL (Figure 6C). A summary of the results in comparison to the Vac phenotype of the ΔM + M_mut_ transformants is presented in Figure 5B. Overall, 22 of these substitutions resulted in a Vac^–^ or Vac^±^ phenotype, and eight of these yielded in an altered fluorescence indicative of an altered M/L interaction. Vac^–^ transformants were obtained when GvpM was altered in HAI (pos. 9–11) or in the GAV motif. In addition, all substitutions in the RAAIA motif lowered the gas vesicle formation, and the M_A45E_ also altered the fluorescence of M_A45E_/L transformants. It is possible that GvpM interacts with GvpL *via* GAV/RAAIA, and that an altered interaction of M_mut_/L contributed to or even caused the Vac^±^ or Vac^–^ phenotype.

### Interaction of GvpJ With GvpM and GvpA as Well as Other Accessory Gvp

**FIGURE 7:**
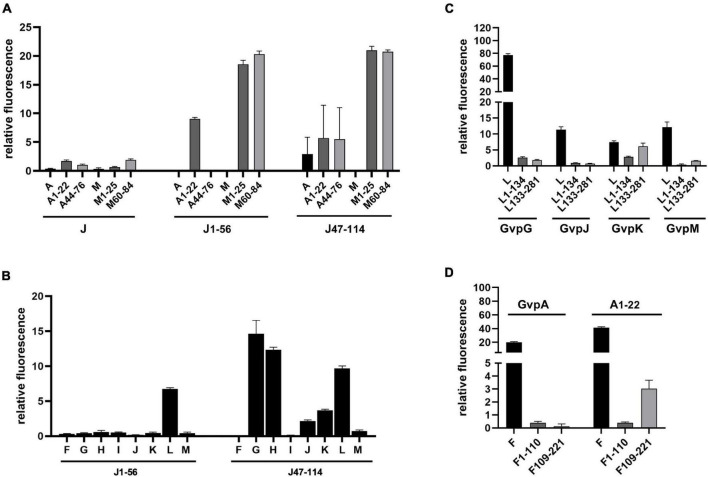
Split-GFP analyses of Gvp proteins. **(A)** Rf values of the interaction studies of GvpJ, J1–56, or J47–114 with the entire GvpA and GvpM, or with N- and C-terminal fragments. The fragments indicated at the bottom are A1–22 and A44–76 in the case of GvpA, M1–25, and M60–84 in the case of GvpM, and J1–56 and J47–114 in the case of GvpJ. **(B)** Rf values of the interaction study of J1–56 and J47–114 with the accessory proteins GvpF through GvpM. **(C)** Rf values of the interaction study of L1–134 or L133–281 with GvpG, GvpJ, GvpK, and GvpM. **(D)** Rf values of the interaction study of F1–110 and F109–221 with GvpA and A1–22.

Split-GFP analyses were also performed to determine whether GvpA, GvpJ, and GvpM are able to interact. We used either the entire proteins, or N-terminal or C-terminal fragments to circumvent unspecific aggregations due to the hydrophobic central portions of these proteins that interfere with the assembly of GFP. The fragments A1–22 and A44–76 of GvpA (Völkner et al., 2020) exclude the hydrophobic β1-β2 portion, and the fragments M1–25 (M25N) and M60–84 (M25C) (Winter et al., 2018) exclude the hydrophobic central portion of GvpM (see Figure 5B). The fragments J1–56 and J47–114 of GvpJ contained the β1-β2 region in J1–56, and both fragments overlapped by 10 aa (Figure 4A). As already observed by Völkner et al. (2020), a very low fluorescence (rf 0.5) was observed for the interaction of the entire proteins (J/A or J/M), but the fragments of GvpA or GvpM were also unable to interact with the entire GvpJ (rf 2) (Figure 7A). Thus, it is difficult to demonstrate an interaction with the entire GvpJ. Using the fragment J1–56 or J47–114 for similar split-GFP analyses, an interaction was not observed with the entire GvpA or GvpM. However, a high fluorescence (rf 20) was obtained when J1–56 or J47–114 were tested for an interaction with the N- or C-terminal fragment of GvpM (Figure 7A). The J1–56 fragment also interacted with A1–22 (rf 10), suggesting that GvpJ is able to contact the N-terminal aa including α1 of GvpA. The interaction of the C-terminal fragment J47–114 with both fragments of GvpA yielded a lower rf value (rf 5) (Figure 7A). These results underlined that GvpA and GvpJ are able to interact. Since the two GvpM and the two GvpA fragments lack the hydrophobic central portion of these proteins, the interaction did not depend on the β1–β2 sequences of both proteins, or on the RAAIA motif of GvpM, but involves the hydrophilic N-terminal (including α1) or C-terminal portions of GvpA or GvpM.

Fragments J1–56 and J47–114 were also used to test for interactions with the accessory proteins GvpF through GvpM in X/Jfrag (X = F, G, H, I, J, K, L, and M) transformants. In case of J1–56, only GvpL was detected as interaction partner (rf 6.7) (Figure 7B). In case of the hydrophilic J47–114, a high fluorescence (rf > 10) was observed with transformants containing GvpG, GvpH, or GvpL, implying that these proteins interact with J47–114 (Figure 7B). An interaction was not detectable with GvpF or GvpI, whereas GvpJ, GvpK, and GvpM yielded low rf values (rf < 5) (Figure 7B). The data are summarized in Table 2, and the original data are presented in Supplementary Table 4. Overall, the results suggested that GvpG, GvpH, and GvpL are interaction partners of GvpJ and able to bind to the C-terminal half of GvpJ. The results supported previous data obtained with CBD-tagged Gvp (Völkner et al., 2020).

**TABLE 2:** Interactions determined for GvpM, GvpJ, and GvpL.

Gvp	Interaction determined	
Fragment	Protein	Fragment
M1–25	GvpL (rf 55)*	J1–56 (rf 20)
		J47–114 (rf 20)
M60–84	GvpF (rf 12)*	J1–56 (rf 20)
	GvpH (rf 12)*	J47–114 (rf 20)
	GvpL (rf 12)*	
J1–56	GvpL (rf 20)	A1–22 (rf 10)
		M1–25 (rf 20)
		M60–84 (rf 20)
J47–114	GvpG (rf 15)	A1–22 (rf 5)
	GvpH (rf 13)	A44–76 (rf 5)
	GvpL (rf 11)	M1–25 (rf 20)
		M60–84 (rf 20)

*Interaction determined by Winter et al. (2018).

### Interaction of Accessory Gvp With GvpL, and of GvpA With GvpF

The 32-kDa GvpL and the 23-kDa GvpF are the largest accessory proteins and 35% related at the sequence level, but the structural model of both proteins is almost identical except for a loop region in GvpL (Winter et al., 2018). A 3D crystal structure of GvpF derived from the cyanobacterial gas vesicle producer *Microcystis aeruginosa* is available (Xu et al., 2014) and was used to obtain the homology model structures of the haloarchaeal GvpF and GvpL (Winter et al., 2018). Both Gvp proteins exhibit two domains. GvpF is the only interaction partner of GvpA, whereas GvpL appears to be the only interaction partner of GvpJ and GvpM as determined by split-GFP (Völkner et al., 2020). Both proteins interact with other accessory Gvp (GvpF with L, H, I, and G; GvpL with F, G, H, I, and K).

Fragments of GvpF and GvpL separating the two domains (F1–110 and F109–221; L1–134 and L133–281) were used for split-GFP analyses to localize the interaction site of GvpA in GvpF, and of GvpJ or GvpM in GvpL more precisely. In addition, the interaction of G, J, K, and M was studied with the fragments of GvpL (Figure 7C), since all these proteins interact with GvpL (Völkner et al., 2020). GvpK interacted with L133–281 (rf 6.1) in a similar range as determined for the K/L interaction (rf 7.4). However, the other three Gvp did not interact with the two GvpL fragments (rf < 2.6), whereas the entire GvpL interacted with these proteins (Figure 7C). The G/L interaction yields the highest rf value (rf 77.45) determined for all Gvp interactions studied so far (Völkner et al., 2020 and this report); nevertheless, the two fragments of GvpL did not contact GvpG. It appears that an intact 3D structure of GvpL is required for the G/L, J/L, and M/L interactions in the region where GvpL was split into two fragments. Similar analyses were performed with the related GvpF and its interaction partner GvpA. The entire GvpF and the two fragments F1–110 and F109–221 were tested with GvpA and fragment A1–22 as interaction partner (Figure 7D). An interaction was only observed with A/F (rf 20) and A1–22/F (rf 41), i.e., with the entire GvpF protein. The rf values observed with the two GvpF fragments were low (rf < 4), indicating that an intact GvpF structure was required around the split site to contact GvpA or the first 22 aa of GvpA.

## Discussion

**FIGURE 8:**
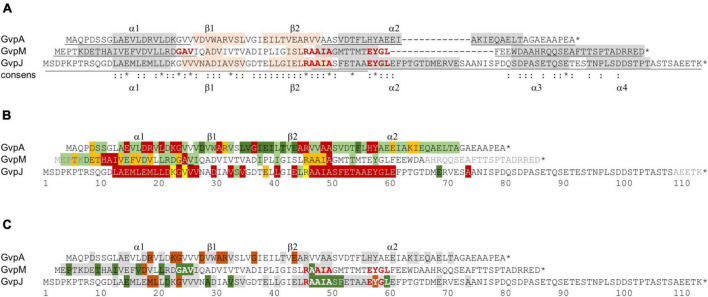
Comparison of the results on GvpA, GvpJ, and GvpM. **(A)** Alignment highlighting the structural features of the three proteins. The α-helical regions predicted are shaded in gray and the β-sheet regions in pink. The sequence motifs GAV, RAAIA, and EYGL are marked in red letters. Underlined are the sequences present in the N- or C-terminal fragments of GvpA, GvpJ, and GvpM used in our studies. The consensus is marked on the bottom: *, three identical residues; two identical aa. **(B)** Alignment comparing the results on the Vac phenotype in the respective ΔX + X_mut_ transformants (X = A, J, or M). Vac^+^ transformants are shaded in green (wild-type gas vesicles); dark green indicates that a substitution by alanine yields cylinder-shaped gas vesicles; orange indicates that a substitution yields Vac^±^ transformants. Residues where a substitution leads to a Vac^–^ phenotype are shaded in red. Substitutions in GvpJ leading to unstable gas vesicles are shaded in yellow. The residues near the N- or C-terminus shown in gray could be deleted without an effect on gas vesicle phenotype. Residues not shaded were not explored. **(C)** Comparison of the results on the A/F interaction (Völkner et al., 2020), and of the J/L and M/L interaction studies presented in this report. The aa shaded in green leads to a higher fluorescence in the transformants, whereas aa shaded in dark red yielded a lower fluorescence when substituted (mostly by alanine). The residues shaded in gray yielded a similar GFP fluorescence as the wild-type proteins GvpA, GvpJ, or GvpM. Residues not shaded were not investigated. The star at the end of the sequences marks the end of each amino acid sequence.

The two accessory proteins GvpJ and GvpM are related to the major gas vesicle structural protein GvpA and grouped in the A-J-M family of hydrophobic gas vesicle proteins. The similarity ranges from 48% (A-M) to 60% (J-M) and is confined to the first 60 aa of these proteins exhibiting the predicted α–β–β–α structure (Figure 8A and Supplementary Figure 1). The functions of GvpM and GvpJ during gas vesicle formation are not known so far; both are encoded by the *gvpFGHIJKLM* gene cluster co-transcribed at the beginning of gas vesicle formation. Single amino acid (aa) substitutions or small deletions at the N- or C-terminus were introduced in both proteins, and the respective variants were studied in *Hfx. volcanii* ΔJ + J_mut_ or ΔM + M_mut_ transformants for gas vesicle formation. In addition, the variants were applied in J_mut_/L or M_mut_/L interaction studies to determine the aa involved in the binding more precisely.

### Mutations in GvpJ and GvpM Have Different Effects on Gas Vesicle Formation

GvpJ with 114 aa is the largest protein of the A-J-M family and contains a unique and hydrophilic sequence of 41 aa at the C-terminus. An alignment of different GvpJ sequences from other archaeal or bacterial gas vesicle producers indicates that only the N-terminal α–β–β–α portion of GvpJ is conserved, but not the last 41 aa at the C-terminus; even the GvpJ protein encoded by the c-vac region of *Hbt. salinarum* or by the related mc-vac region of *Hfx. mediterranei* (Englert et al., 1992) is distinct from GvpJ derived from p-vac investigated here. These observations suggest that this portion of GvpJ is even *gvp* gene-cluster specific. This unique C-terminal portion turned out to be important for the function of GvpJ. Already a deletion of five aa yielded Vac^–^ ΔJ + J_Δ5C_ transformants, indicating that an alteration in size was not possible without losing the function of GvpJ. In contrast, the last 25 non-conserved aa of GvpM can be deleted without affecting gas vesicle formation (Winter et al., 2018).

A total of 66 substitutions were introduced to alter the predicted α–β–β–α region of GvpJ (Supplementary Table 2), and 56 of the resulting ΔJ + J_mut_ transformants were Vac^–^ (Figure 8B). Almost all substitutions in α1 yielded Vac^–^ transformants, and single substitutions in the β-sheet region or all substitutions within pos. 47–61 including part of α2 as well. The related α2 in the structural model of GvpA is not that important; many GvpA variants with a substitution in α2 (especially in the second half) yield Vac^+^ ΔA + A_mut_ transformants (Knitsch et al., 2017; Figure 8B). The RAAIA motif located between β2 and α2 is only present in GvpJ and GvpM; GvpA contains the related RVVAA sequence at this position (Figure 8A). An alteration of the arginine in RAAIA of GvpJ yielded unstable gas vesicles in ΔJ + J_R46A_ transformants, but all other substitutions prevented their formation. In contrast, most alterations in RAAIA of GvpM only reduce the number of gas vesicles per cell (Figure 8B; Tavlaridou et al., 2014). Alterations in RVVAA of GvpA result in Vac^–^ transformants when the arginine or the two alanine residues are substituted, whereas the valine–alanine substitutions resulted in Vac^+^ ΔA + A_mut_ transformants (Knitsch et al., 2017; Figure 8B). Overall, it appears that RAAIA is important for the function of GvpJ and GvpM. The sequence is exposed at the surface and might constitute a binding site for other Gvp such as GvpL (see below).

Only ten out of sixty-six GvpJ substitution variants yielded a Vac^+^ or Vac^±^ phenotype when tested in ΔJ + J_mut_ transformants, indicating that the predicted α–β–β–α structure of GvpJ is sensitive to alterations. It is interesting to note that three of the ΔJ + J_mut_ transformants (ΔJ + J_K23A_, J_V25A_, or J_R46A_) contained gas vesicles that were impossible to isolate by flotation. The alterations did not prevent the formation of the gas-filled structures, but the wall might be less stable to withstand an increase in the pressure. The critical pressure resulting in the collapse of the haloarchaeal gas vesicles is 0.09 MPa (Walsby, 1994). This phenomenon was only observed with ΔJ + J_mut_ transformants; none of the gas vesicles formed by ΔA + A_mut_ or ΔM + M_mut_ transformants exhibited this feature (Knitsch et al., 2017, and this report). This result argues that GvpJ is part of the gas vesicle wall. Overall, the α–β–β–α structure of GvpJ is vulnerable, whereas most of the substitutions in GvpM only reduced the number of gas vesicles or had no effect (Figure 8B). An alteration of the shape of gas vesicles, as often observed with GvpA variants, was only found with the variants J_D22K_ and J_E69A_ that produced somewhat larger cylinder-shaped gas vesicles.

GvpM with 84 aa is smaller than GvpJ. Many of the 38 ΔM + M_mut_ transformants contained gas vesicles of wild-type shape (16 Vac^+^, 12 Vac^±^) (Table 1). Only ten were Vac^–^, which is much less compared to the large fraction of Vac^–^ ΔJ + J_mut_ transformants. We mutated especially the first 25 aa including α1 of GvpM to define the impact of this region on gas vesicle formation and analyzed the interaction of GvpL with the M1–25 fragment since previous analyses suggested that GvpL binds predominantly to this region (Winter et al., 2018). Several substitutions in α1 or in the adjacent GAV motif of GvpM did not affect gas vesicle formation (Figure 8B). Vac^–^ transformants only occurred when an aa of 9-HAI-11 or the alanine in the GAV motif was substituted. Alterations in GAV also increased the fluorescence of the respective M1–25_mut_/L transformants (Figure 6A), suggesting that GAV rather than HAI might be the GvpL contact site. Overall, helix α1 of GvpM was less important compared to α1 of GvpJ or GvpA (Figure 8B). It is interesting to note that none of the aa substitutions in α1 of GvpM, GvpJ, or GvpA affected gas vesicle formation in the same way in all three proteins despite the conserved aa sequence. For example, a Vac^±^ phenotype was observed when non-polar aa in α1 of GvpM were substituted by alanine, whereas substitutions of the conserved polar aa always resulted in Vac^+^ ΔM + M_mut_ transformants. In contrast, alterations of non-polar aa in α1 of GvpA have no effect, whereas a substitution of polar aa often results in Vac^–^ ΔA + A_mut_ transformants, suggesting that mainly salt bridges are formed between α1 and its interaction partner(s) (Knitsch et al., 2017). These observations also imply that GvpA and GvpM cannot interact *via* α1, whereas GvpJ and GvpA might interact *via* the polar aa in α1. Any alteration in α1 of GvpJ resulted in Vac^–^ transformants.

Overall, the effect of mutations in GvpA, GvpJ, or GvpM on gas vesicle formation were quite different, although similar substitutions were done especially in the conserved regions α1 and α2 of the three proteins. The results underline that the three Gvp proteins have distinct functions and cannot substitute each other. GvpJ appears to have a central function during gas vesicle formation. The protein is present in any gas vesicle gene cluster of bacteria and archaea, whereas GvpM is haloarchaea-specific. Nevertheless, GvpM is essential for gas vesicle formation. GvpJ and GvpM are produced in early stages of gas vesicle assembly, and most likely involved in the formation of the two conical end caps. GvpM might participate in very early steps, since gas vesicles of wild-type shape are either formed or are absent in ΔM + M_mut_ transformants. GvpJ is presumably involved in the formation of the wall, since alterations in the gas vesicle shape and strength occurred with certain variants. GvpA is the major gas vesicle protein and constitutes > 95% of the gas vesicle wall (Walsby, 1994). GvpA aggregates into a helix of low pitch running perpendicular to the long axis of the gas vesicle and thus contacts mainly GvpA molecules within the ribs and also between the ribs, but also contacts GvpJ during early stages of growth.

### Fragments of GvpJ Interact With Fragments of GvpA or GvpM

So far, interactions of GvpA, GvpJ, and GvpM were not detectable by split-GFP, presumably due to the hydrophobic nature of these proteins leading to unspecific aggregations that interfere with the assembly of GFP. However, an interaction has been observed by tagging one of these proteins with the cellulose binding domain, CBD, and selecting the binding partner *via* a cellulose matrix (Völkner et al., 2020). To circumvent the aggregation problem and to confine the interaction sites, fragments of GvpA, GvpJ, and GvpM were used for an interaction study by split-GFP. The N- and C-terminal fragments of GvpM (M1–25, M60–84) (Winter et al., 2018) and GvpA (A1–22, A44–76) (Völkner et al., 2020) exclude the central hydrophobic portions, whereas the two fragments of GvpJ (J1–56 and J47–114) overlapped for 10 aa in the center (see Figure 8A).

The N-terminal J1–56 fragment encompasses α1–β1–β2 up to the EYGL motif and also includes the RAAIA motif (Figure 4A). This fragment interacted with M1–25, but also with A1–22 (Figure 7A) implying that the α1 helices of GvpA and GvpM interact with the N-terminal portion of GvpJ. The contact might involve α1 in all cases. It is possible that the non-polar aa are involved in the M/J interaction (because alterations of these aa in GvpM reduce gas vesicle formation), whereas the polar aa of GvpA participate in the A/J (and A/A) interaction. This would explain why all aa found in α1 of GvpJ were essential and substitutions always resulted in Vac^–^ ΔJ + J_mut_ transformants. Fragment J1–56 also bound to M60–84, but since ΔM + M_Δ25C_ transformants are Vac^+^, these sequences can be deleted without affecting the gas vesicle formation (Winter et al., 2018). The C-terminal fragment J47–114 starts at AAIA of the RAAIA motif and contains besides α2 the unique C-terminal portion of GvpJ. This fragment interacted with M1–25 and M60–84, and also with A1–22 and A44–76, implying that two contact sites each are present in both proteins. The unique region of GvpJ is important since a deletion of the last five aa already affected gas vesicle formation. An interaction was not observed when the entire GvpA, GvpJ, and GvpM proteins were applied; it appears that the presence of the hydrophobic β1–β2 portions are indeed the reason why the J/A or J/M interactions were not detectable by split-GFP (Völkner et al., 2020, and this report). The two fragments of GvpM exclude the β1–β2–α2 portion including the RAAIA and EYGL motif, demonstrating that these sequences are not required for the J/M interaction.

Overall, we could show that GvpJ is able to interact with both fragments of GvpM and also contacts the N-terminal region (α1) of GvpA with its N-terminal portion. The hydrophobic portions of GvpM and GvpA are not involved in these contacts. The β1–β2 sheets of GvpA presumably constitute the gas-facing surface of the gas vesicle wall, whereas α1 and α2 of GvpA mediate the A/A interaction within the ribs and/or between the ribs of the wall (Knitsch et al., 2017), or serve as contact sites for other Gvp proteins. We could show that both α-helices of GvpA contact GvpJ, and A1–22 also harbors the contact site of GvpF (Völkner et al., 2020). Thus, GvpF is not the only interaction partner of GvpA as suggested earlier. The 23.9-kDa GvpF and the 32-kDa GvpL are able to bind the A-J-M proteins also in presence of the hydrophobic β1–β2 region.

### Interaction of GvpJ and GvpM With GvpL

**FIGURE 9:**
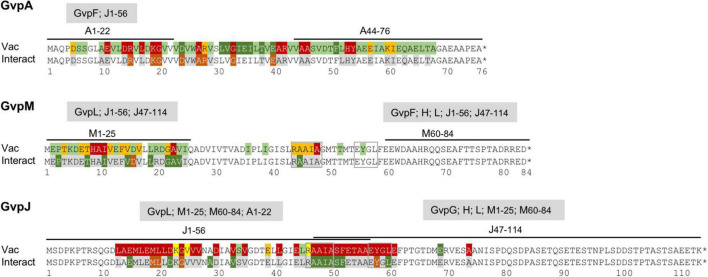
Overview of the results obtained for the GvpA, GvpM, and GvpJ proteins in this report. The alterations leading to a Vac phenotype are compared to the results on the interaction studies, and the interaction partners of the fragments applied in the split-GFP analyses are indicated on top of the respective sequence. The colors used for shading the aa residues in each sequence are the same as in Figure 8.

GvpL was the only Gvp that interacted with the entire GvpJ or GvpM when analyzed by split-GFP (Völkner et al., 2020). The substitution variants of GvpJ or GvpM were used to determine the effect of the mutations on the J_mut_/L or M_mut_/L interaction by split-GFP. In case of GvpJ, a negative effect was observed on the interaction when the GvpJ variant contained a substitution at the end of α1 and in loop1 (M19, L20, G24), or in the EYGL motif of α2 (Y58) (Figure 9). A significant increase in the fluorescence was observed with the variants containing a substitution in the AAIA motif or the adjacent Ser and Phe, implying that this region (pos. 47–52) influences the interaction (Figure 8C). The C-terminus of GvpJ was not involved, since the J_Δ5C_ through J_Δ30C_ variants showed no difference (Figure 3B). Thus, AAIASF and EYGL might be involved in the J/L interaction. Since the variants leading to an altered fluorescence all yielded Vac^–^ ΔJ + J_mut_ transformants, an altered J_mut_/L interaction could have contributed to the lack of gas vesicles (Figure 9).

In the case of GvpM, the interaction with GvpL was studied using variants of fragment M1–25 mainly consisting of α1 and part of loop 1. Previous results already demonstrated that GvpL interacts with M1–25 (Winter et al., 2018). Similar aa substitutions were introduced in M1–25 as already tested with GvpM for gas vesicle formation in ΔM + M_mut_ transformants (Figure 5B), and the only reduction in fluorescence of the M1–25_mut_/L transformants was observed with the D16A substitution (Figure 8C). However, ΔM + M_D16A_ transformants are Vac^+^ demonstrating that the D16A substitution does not affect the formation of gas vesicles (Tavlaridou et al., 2014). Only three alterations, I11D (part of HAI in α1), A23D, and V24D (part of GAV in loop 1), yielded an altered fluorescence and also resulted in Vac^–^ ΔM + M_mut_ transformants (Table 1). The GAV motif locates close to the RAAIA motif in the 3D model of GvpM, and this area might contact GvpL.

The J/L and M/L interactions are comparable to the interaction of GvpA with GvpF. Previous analyses of the GvpF binding site in GvpA localized the contact to R15 and K19 in α1, G20 in loop 1, and to aa in the β1–β2 region, but the RVVAA sequence of GvpA is not involved (Figure 8C; Völkner et al., 2020). Except for the substitution of D24 or E40 resulting in cylinder-shaped gas vesicles, a substitution of all other aa yield Vac^–^ ΔA + A_mut_ transformants. In these latter cases, the lack of gas vesicles could be caused by an altered A/F contact (Figure 9). The comparison of the three proteins showed that α1 and loop1 of the A-J-M proteins are involved in the A/F, J/L, and M/L interactions, but the putative contact sites are different.

### Fragments of GvpF or GvpL Are Unable to Interact With the A-J-M Proteins

GvpF and GvpL are important accessory proteins, and genes encoding at least one of these proteins are found in all bacterial or archaeal gas vesicle gene clusters analyzed so far. A crystal structure of GvpF derived from the cyanobacterium *M. aeruginosa* is available (published as GvpF, but the sequence shows a higher similarity to GvpL) (Xu et al., 2014; Supplementary Figure 7). Homology modeling reveals a structural model of the haloarchaeal GvpF and GvpL implying two domains (Winter et al., 2018). The two fragments used in our interaction studies comprised these two domains (F1–110 and F109–221; L1–134 and L133–281). In the case of GvpF, the A/F interaction is confined to the N-terminal fragment A1–22 (Völkner et al., 2020). However, neither GvpA nor A1–22 was able to interact with F1–110 or F109–221 (Figure 7D), suggesting that the native GvpF structure near the split site is required for the A/F interaction. A similar result was observed with GvpL, where only the entire GvpL bound GvpJ or GvpM, and interactions with the fragments L1–134 or L133–281 were not detectable (Figure 7C). Thus, the A/F or J/L and M/L interactions rely on the native structure around the split in GvpL or GvpF. Since the interactions of the A-J-M proteins are only observed with GvpF and GvpL by split-GFP, both proteins might act as “chaperones,” supporting the desired aggregations at the start of the gas vesicle assembly. Preventing unspecific aggregations of the A-J-M proteins and bringing together additional Gvp could be the function of GvpL that also binds GvpF, GvpG, GvpH, GvpI, and GvpK (Völkner et al., 2020; and this report). Similar to GvpJ and GvpM, GvpG required the entire GvpL for binding, whereas GvpK bound the C-terminal portion L133–281 (Figure 7B). Further analyses are required to define the binding sites and also the function of GvpF and GvpL in more detail.

### The C-Terminal Fragment of GvpJ Contacts Other Accessory Gvp

Our mutation analyses suggested that GvpJ plays a central role in the assembly of the gas vesicle wall and contacts other Gvp. While the hydrophobic N-terminal fragment J1–56 interacted only with GvpL, the hydrophilic C-terminal fragment J47–114 interacted with GvpG, GvpH, and GvpL, but not with GvpF or GvpI, and weak interactions (rf < 5) were observed with GvpJ (dimer), GvpK, and GvpM (Figure 7B). The weak interaction of J47–114 with GvpJ or GvpM was expected, since unspecific aggregations of these two hydrophobic proteins interfere with the assembly of GFP in the split-GFP analyses. GvpG, GvpH, and GvpL are already known as interaction partners of GvpJ; all of them are able to select GvpJ when tagged with CBD (Völkner et al., 2020). Here, we could show that the C-terminal portion of GvpJ mediates these interactions. It is possible that the conserved AAIA motif and/or α2 region is involved, since all of these aa are essential (Figure 9). Whether GvpG, GvpH, and GvpL bind at the same time or interact sequentially with GvpJ is currently unknown. Also, the function(s) of these complex(es) in gas vesicle formation remains to be investigated.

## Conclusion

Here, we explored the effect of mutations in the homologous accessory protein GvpJ or GvpM on the assembly of gas vesicles and also on the interactions with their partner protein GvpL or other accessory Gvp proteins. GvpJ appears to be very sensitive to alterations (56 of 66 mutations result in a Vac^–^ phenotype), whereas 2/3 of the mutations in GvpM had no effect. Our findings highlight the importance of GvpJ and GvpM, but many questions remain unanswered. GvpJ and GvpM are produced together with other accessory proteins from the *gvpFGHIJKLM* transcript at the beginning of gas vesicle assembly, and all of these Gvp proteins interact. The major gas vesicle structural protein, GvpA, binds GvpF, and GvpJ and GvpM both interact with GvpL that also binds GvpF. GvpL also interacts with all other accessory Gvp proteins and might bring all of them in close contact. It will be interesting to determine the interaction sites of the accessory proteins in GvpL more precisely and unravel their role in the initial aggregation steps in further detail.

## Data Availability Statement

The original contributions presented in the study are included in the article/Supplementary Material, further inquiries can be directed to the corresponding author/s.

## Author Contributions

RK, AJ, KV, and FP planned the study. RK, AJ, and KV performed the analysis. RK performed the studies on ΔJ + J_mut_ transformants. KV performed those on ΔM + M_mut_ transformants including the M/L interaction studies by split-GFP. AJ performed the J/L interaction studies and the additional interaction studies described. FP and AJ wrote the manuscript. All authors discussed the results and approved the final version.

## Conflict of Interest

The authors declare that the research was conducted in the absence of any commercial or financial relationships that could be construed as a potential conflict of interest.

## Publisher’s Note

All claims expressed in this article are solely those of the authors and do not necessarily represent those of their affiliated organizations, or those of the publisher, the editors and the reviewers. Any product that may be evaluated in this article, or claim that may be made by its manufacturer, is not guaranteed or endorsed by the publisher.
