# Supplementary material for: Effect of Mutations in GvpJ and GvpM on Gas Vesicle Formation of Halobacterium salinarum
Source: Front Microbiol. 2021 Dec 16;12:794240. doi: 10.3389/fmicb.2021.794240 (PMC8716928; doi:10.3389/fmicb.2021.794240)
Supplement: Supplementary file 1 [file Data_Sheet_1.pdf]

Supplementary Figures and Tables

Effect of Mutations in GvpJ and GvpM on Gas Vesicle Formation of *Halobacterium salinarum*

Alisa Jost[1], Regine Knitsch[1], Kerstin Völkner[1], and Felicitas Pfeifer[1*]

[1]Microbiology and Archaea, Department of Biology, Technical University Darmstadt, Darmstadt, Germany

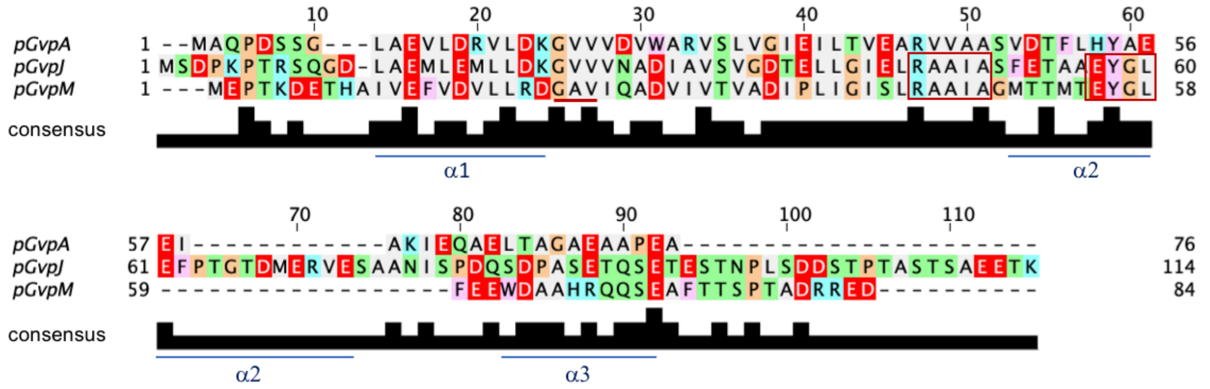

Supplementary Figure S1. Alignment of the aa sequence of GvpA, GvpJ and GvpM derived from the p-vac region of *Halobacterium salinarum* PHH1.The highlighted sequence motifs of GvpJ and GvpM (GAV, RAAIA, EYGL) are discussed in the text. The consensus of the three sequences depicted by black beams is shown below. Predicted α-helices of GvpJ are labelled α1 through α3.

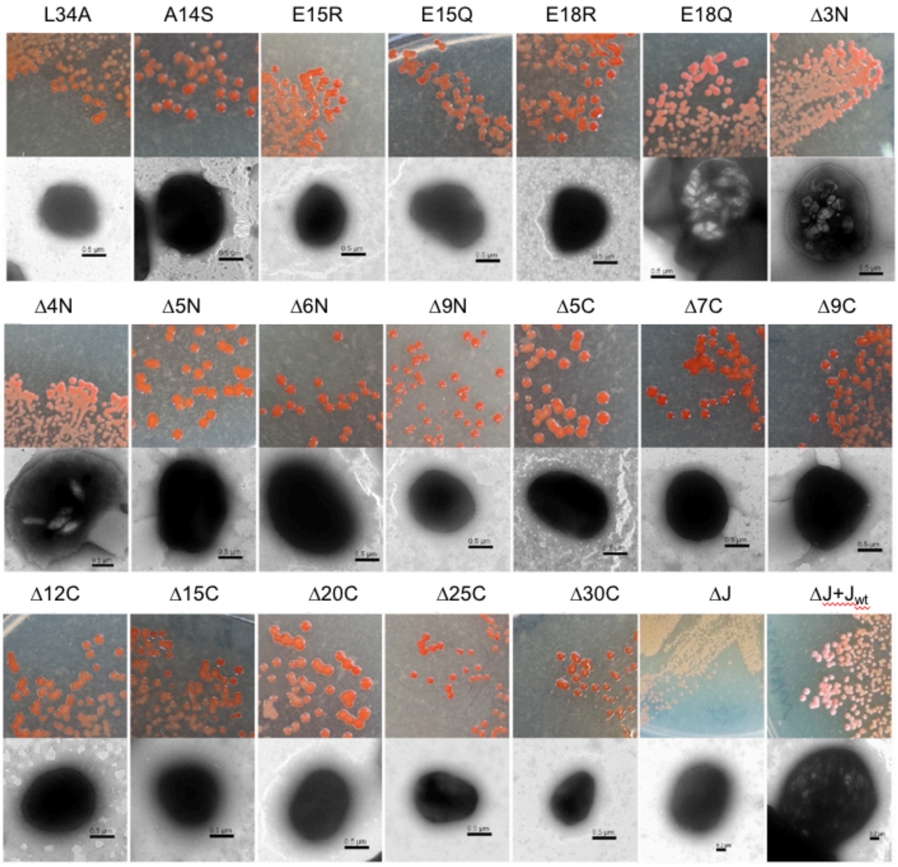

Supplementary Figure S2. Colonies of $\Delta J+J_{wt}$ and $\Delta J+J_{mut}$ transformants on solid media (top), and cells analyzed by transmission electron microscopy (underneath). The respective GvpJ variant is labelled on top. Colonies of gas vesicle containing cells are turbid and pink (see $\Delta J+J_{wt}$ bottom row, right). The red color is due to the caroteine bacterioruberin. In contrast, colonies of Vac^{-} cells are transparent. The difference in the darkness of the red color depends on the oxygen supply during growth. Cells of several colonies were inspected by transmission electron microscopy for the possession of gas vesicles. In each case, a representative cell is shown underneath.

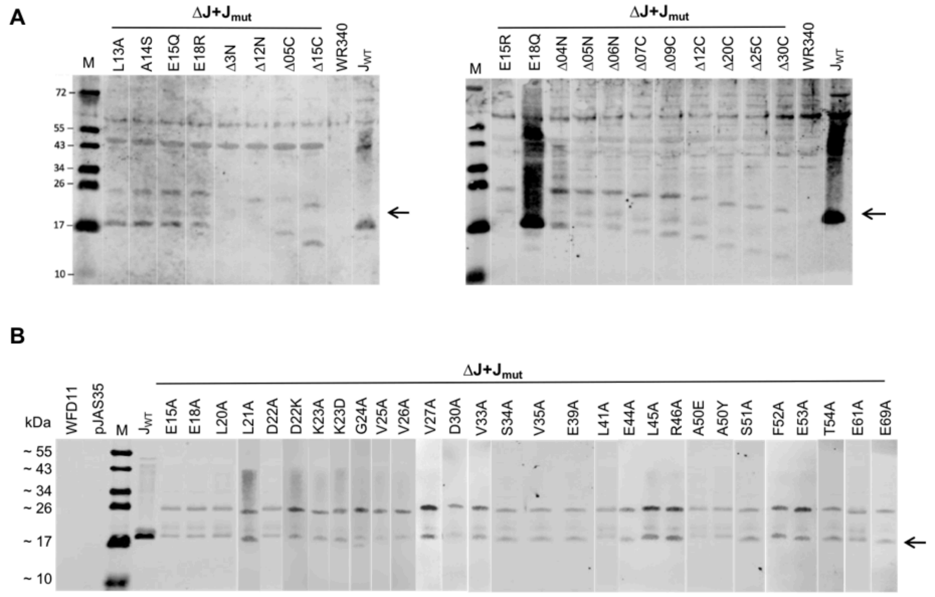

Supplementary Figure S3. Western analyses to determine the presence of GvpJ. Total proteins were isolated from ΔJ+J$_{wt}$ or ΔJ+J$_{mut}$ transformants, and 20 µg of proteins were separated by SDS-PAGE, followed by transfer on a PVDF membrane and treatment with an antiserum raised against GvpJ. The respective substitution- or deletion variant is indicated on top. Arrows on the right mark the position of GvpJ.

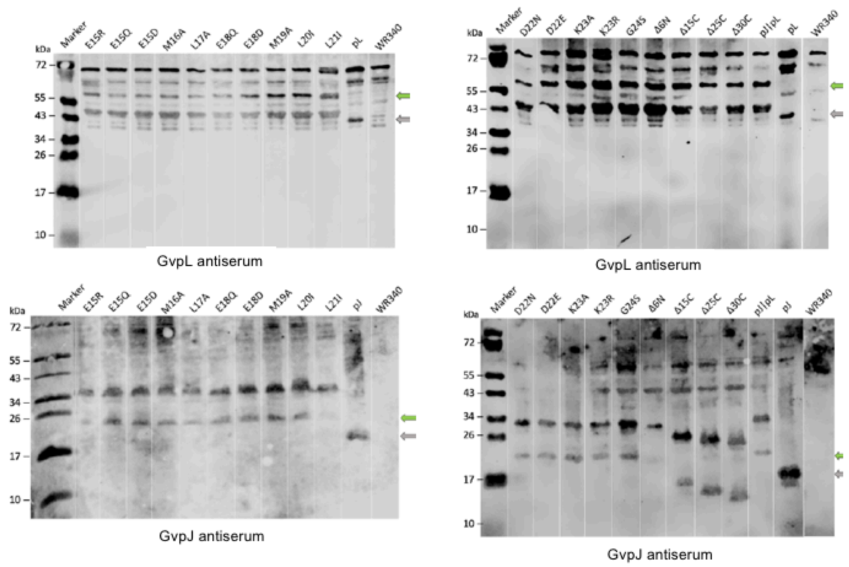

Supplementary Figure S4. Western analysis to determine the presence of GvpJ and GvpL in the interaction study. Total proteins were isolated from cells containing J$_{mut}$CGFP and NGFPL, and 20 µg of proteins were separated by SDS-PAGE, followed by transfer to a PVDF membrane and treatment with the respective antiserum to detect NGFPL (top) or JCGFP (bottom). Lysates of *Hfx. volcanii* transformants expressing *gvpJ*-pJAS35 or *gvpL*-pJAS35 were used as control. Green arrows mark the position of the expected Gvp-GFP fusion protein, grey arrows the original GvpJ or GvpL.

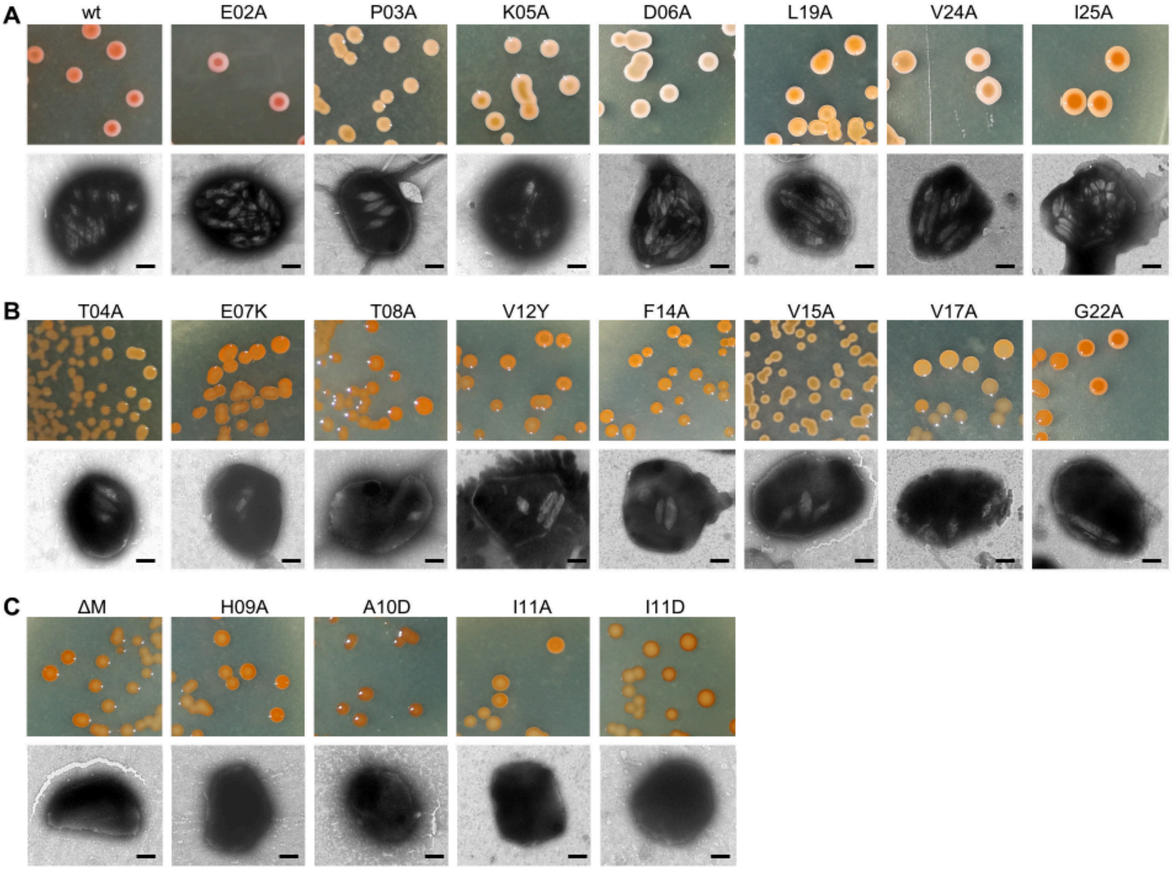

Supplementary Figure S5. Colonies of $\Delta M+M_{wt}$ and $\Delta M+M_{mut}$ transformants on solid media (top), and representative cells analyzed by transmission electron microscopy for the possession of gas vesicles. The respective GvpJ variants are labeled on top. **(A)** Transformants producing gas vesicles similar to wild type (wt) (Vac^+ phyenotype). **(B)** Transformants containing a few gas vesicles only ($Vac^{\pm}$). **(C)** Transformants exhibiting a Vac^- phenotype. Further explanations are given in the text.

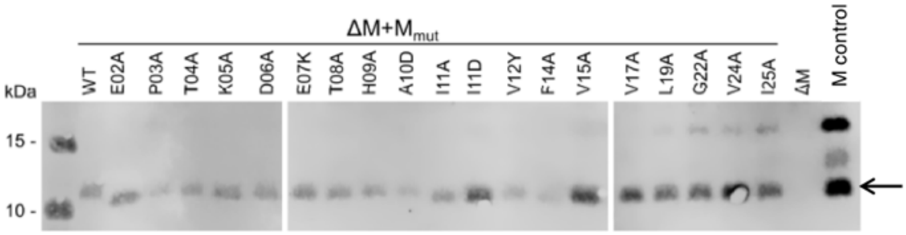

Supplementary Figure S6. Western analyses to determine the presence of GvpM. Total proteins were isolated from $\Delta M+M_{wt}$ or $\Delta M+M_{mut}$ transformants, and 20 µg of proteins were separated by SDS-PAGE followed by transfer on a PVDF membrane and treatment with an antiserum raised against GvpM. The respective substitution of deletion variant is indicated on top. The arrow on the right marks the position of GvpJ.

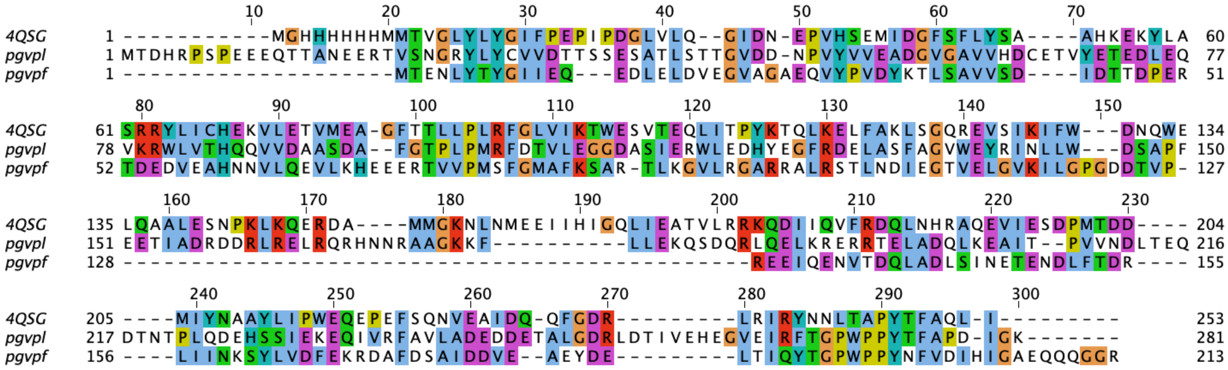

Supplementary Figure S7. Alignment of GvpF derived from the cyanobacterium *Microcystis aeruginosa* and the GvpF and GvpL proteins of the haloarchaeon *Halobacterium salinarum*.

Supplementary Table 1. Oligonucleotides used in this study.

Name	Oligonucleotide sequence (5‘-3‘)*
**p-*gvpM* substitutions in M^{Ex}**	
f-pM/E02A	atgg*ca*ccaacaaaagacgag
f-pM/P03A	atggag*g*caacaaaagacgag
f-pM/T04A	atggagcca*g*caaaagacgag
f-pM/K05A	atggagccaaca*gc*agacgag
f-pM/D06A	atggagccaacaaaag*c*cgag
f-pM/E07K	gac*a*agacacacgcgatcgtt
f-pM/T08A	gacgag*g*cacacgcgatcgtt
f-pM/H09A	gacgagaca*gc*cgcgatcgtt
f-pM/A10D	gacgagacacacg*ac*atcgtt
f-pM/I11A	gcg*gc*cgttgagttcgt
f-pM/I11D	gcg*ga*cgttgagttcgt
f-pM/V12Y	gcgatc*ta*tgagttcgt
f-pM/F14A	gcgatcgttgag*gc*cgt
f-pM/V15A	ttcg*c*cgacgtgttactgc
f-pM/V17A	ttcgtcgacg*c*gttactgc
f-pM/L18A	ttcgtcgacgtg*gc*actgc
f-pM/L19A	ttcgtcgacgtgtta*gc*gc
f-pM/G22A	gacg*c*agccgtgattcaag
f-pM/V24A	gacggagccg*c*gattcaag
f-pM/I25A	gacggagccgtg*gc*tcaag
r-pM/E02, P03, T04, K05, D06	ctgcagagttcggcttcc
r-pM/E07, T08, H09, A10	ttttgttggctccatctgcagag
r-pM/I11, V12, F14	gtgtgtctcgtcttttgttggctc
r-pM/V15, V17, L18, L19	ctcaacgatcgcgtgtgtctc
r-pM/G22, V24, I25	gcgcagtaacacgtcgac
f-pM/D28G	aagcgg*g*cgtgatcgt
r-pM/D28G	gaatcacggctccgtc
f-pM/T32K	gtgatcgtga*a*ggtcgccg
r-pM/T32K	gtccgcttgaatcacggctc
**p-*gvpM* substitutions in split-GFP vector $pM(25N)_N$**	
pM_25N-E02A_fwd	atgcccatgg*c*gccaacaaa
pM_25N-E02A_rev	cactgcagagttcggcttccga
pM_25N-P03A_fwd	atgcccatggag*g*caacaaaa
pM_25N-T04A_fwd	atgcccatggagcca*g*caaaag
pM_25N-K05A_fwd	gccaaca*gc*agacgagacac
pM_25N-K05A_rev	tccatgggcatcactgcagag
pM_25N-D06A_fwd	gccaacaaaag*ca*gagacacacg
pM_25N-E07A_fwd	gccaacaaaagacg*c*gacacac
pM_25N-T08A_fwd	gccaacaaaagacgag*g*cacacg
pM_25N-H09A_fwd	cgagaca*gc*cgcgatcg
pM_25N-H09A_rev	tcttttgttggctccatgggcatc
pM_25N-A10D_fwd	cgagacacac*tg*gatcgttgag
pM_25N_I11A_fwd	acacgcg*gca*gttgagttc
pM_25N_I11D_fwd	acacgcg*ga*cgttgagttc
pM_25N_V12Y_fwd	acacgcgatc*ta*tgagttcgtcg
pM_25N_E13A_fwd	acacgcgatcgttg*ca*ttcgtc
pM_25N_I11A_rev	gtctcgtcttttgttggctccatg

3'-BlpI-pM(25N)_D21A	tgttgtt **gctcagc** cc aatcacggctccggc
3'-BlpI-pM(25N)_G22A	tgttgtt **gctcagc** cc aatcacggctgcgtc
3'-BlpI-pM(25N)_A23D	tgttgtt **gctcagc** cc aatcacgtctccgtcgc
3'-BlpI-pM(25N)_V24A	tgttgtt **gctcagc** cc aatcgcggctccgtc
3'-BlpI-pM(25N)_I25A	tgttgtt **gctcagc** cc agccacggctccg
5'-NcoI-pM	tgttgtt **ccatgg** atggagccaacaaaag
3'-BlpI-pM(25N)Δstop	tgttgtt **gctcagc** cc aatcacggctccgtcg

p-*gvpM* substitutions in split-GFP vector pM$_C$

5'-NcoI-pM	tgttgtt **ccatgg** atggagccaacaaaag
3'-BamHI-pMΔstop	agttct **ggatcc** c gtcctctcgccg

p-*gvpJ* substitutions in pBSK(II)+ and pJAS35

5'-PstI-pJ	attc **ctgcag** atg agtgaccccaaaccgac
3'-KpnI-pJ	attc **ggtacc** tca tttggtctcctccgctg
f-pJ/L13A	gcgac*gca*gccgagatg
f-pJ/A14S	gcgacctc*t*ccgagatg
r-pJ/L13, A14	cctgcgagcgcgtc
f-pJ/L13I	*a*tcgccgagatgctggagatgc
f-pJ/A14V	ctcg*t*cgagatgctggagatgctc
f-pJ/A14D	ctcg*a*cgagatgctggagatgctc
r-pJ/L13, A14	gtcgccctgcgagcgcg
f-pJ/E15A	ggcgacctcgccg*c*gatgctggagatg
r-pJ/E15A	catctccagcatc*g*cggcgaggtcgcc
f-pJ/E15R	ctcgcc*ag*gatgctggag
f-pJ/E15Q	ctcgcc*c*agatgctggag
f-pJ/E15D	ctcgccga*t*atgctggagatg
r-pJ/E15	gtcgccctgcgagc
f-pJ/M16A	gag*gc*gctggagatgc
f-pJ/L17A	gagatg*gc*ggagatgctcc
f-pJ/E18R	gagatgctg*c*agatgctccta
f-pJ/E18Q	gagatgctg*ag*gatgctccta
f-pJ/E18D	gagatgctgga*t*atgctcctagac
r-pJ/M16, L17, E18	ggcgaggtcgccc
f-pJ/E18A	gccgagatgctgg*cg*atgctcctagac
r-pJ/E18A	gtctaggagcat*cg*ccagcatctcgg
f-pJ/E18M	*at*gatgctcctagacaaaggagtcgtcg
f-pJ/M19E	gag*ga*gctcctagacaaaggagtcgtc
f-pJ/E18, M19	cagcatctcggcgaggtcgc
f-pJ/M19A	gag*gc*gctcctagacaaagg
f-pJ/L20A	atgctggagatg*gcg*ctagacaaagga
r-pJ/L20A	tcctttgtctag*cgc*catctccagcat
f-pJ/L20I	gagatg*a*tcctagacaaaggagtcgtc
f-pJ/L21I	gagatgctc*a*tagacaaaggagtcgtc
f-pJ/D22N	gagatgctccta*a*acaaaggagtcg
f-pJ/D22E	gagatgctcctaga*a*aaaggagtcgtc
r-pJ/M19, L20, L21, D22	cagcatctcggcgaggtc
f-pJ/L21A	*gc*agacaaaggagtcgtcgtcaac
f-pJ/D22K	cta*a*a*a*aaaggagtcgtcgtcaacgc
f-pJ/K23D	ctagac*gat*ggagtcgtcgtcaacg
f-pJ/D22A	gagatgctcctag*cg*aaaggagtcgtc
r-pJ/D22A	gacgactccttt*cg*ctaggagcatctc

r-pJ/L21, K23	gagcatctccagcatctcggc
f-pJ/K23A	gac*gc*aggagtcgtcgtc
f-pJ/K23R	gac a*g*a gga gtc gtc gtc aac
f-pJ/G24S	gac aaa *agt* gtc gtc gtc aac g
r-pJ/K23, G24	taggagcatctccagcatctcg
f-pJ/G24A	g*c*agtcgtcgtcaacgcggatatcg
f-pJ/V25A	ggag*ca*gtcgtcaacgcggatatcg
f-pJ/V26A	ggagtcg*ca*gtcaacgcggatatcg
f-pJ/V27A	ggagtcgtcg*ca*aacgcggatatcg
r-pJ/G24, V25, V26, V27	tttgtctaggagcatctccagcatctcgg
f-pJ/A29D	*gac*gatatcgcagtcagcgtcgg
f-pJ/D30A	gcgg*c*tatcgcagtcagcgtc
r-pJ/A29, D30	gttgacgacgactcctttgtctaggagc
f-pJ/V33A	g*c*cagcgtcggggacacgg
f-pJ/V33D	g*a*cagcgtcggggacacggaac
r-pJ/V33	tgcgatatccgcgttgacgacg
f-pJ/S34A	*gca*gtcggggacacggaactcctc
f-pJ/V35A	agc*gca*ggggacacggaactcc
r-pJ/S34, V35	gactgcgatatccgcgttgacgacg
f-pJ/E39A	g*c*actcctcggtatcgagttacgggc
f-pJ/L41A	gaactc*gca*ggtatcgagttacgggcc
r-pJ/E39, L41	cgtgtccccgacgctgactgc
f-pJ/E44A	g*c*attacgggccgcgattgcttc
f-pJ/L45A	gag*gc*acgggccgcgattgc
f-pJ/R46A	gagtta*gca*gccgcgattgcttcgttc
r-pJ/E44, L45, R46	gataccgaggagttccgtgtccccgac
f-pJ/R46E	g*ag*gccgcgattgcttcgttc
f-pJ/A47D	cggg*a*cgcgattgcttcgttc
f-pJ/A48D	cgggccg*ac*attgcttcgttc
r-pJ/R46, A47, A48	taactcgataccgaggagttccgtgtc
f-pJ/I49A	*gc*tgcttcgttcgagacagcggc
f-pJ/A50D	attg*a*ttcgttcgagacagcggcc
r-pJ/I49, A50	cgcggcccgtaactcgataccg
f-pJ/A50Y	*ta*ttcgttcgagacagcggccgaatac
f-pJ/A50E	g*aa*tcgttcgagacagcggccgaatac
f-pJ/S51A	gct*g*c*a*ttcgagacagcggccg
r-pJ/A50, S51	aatcgcggcccgtaactcgatacc
f-pJ/F52A	*gc*cgagacagcggccgaatac
f-pJ/E53A	ttcg*c*gacagcggccgaatac
f-pJ/T54R	ttcgaga*g*agcggccgaatacg
r-pJ/F52, E53, T54	cgaagcaatcgcggcccg
f-pJ/T54A	*g*cagcggccgaatacgggctc
r-pJ/T54A	ctcgaacgaagcaatcgcggcc
f-pJ/A55D	*gac*gccgaatacgggctcgag
f-pJ/A56D	gcgg*a*cgaatacgggctcgag
r-pJ/A55, A56	tgtctcgaacgaagcaatcgcg
f-pJ/Y58A	*gca*gggctcgagttcccaacgg
r-pJ/Y58A	ttcggccgctgtctcgaacgaag
f-pJ/E57A	g*ct*tacgggctcgagttcccaac
f-pJ/Y58E	gaa*gag*gggctcgagttcccaac
f-pJ/G59A	gaatac*gct*ctcgagttcccaacgg
r-pJ/E57, Y58, G59	ggccgctgtctcgaacgaagc
f-pJ/L60A	*gct*gagttcccaacggggacg
r-pJ/L60A	cccgtattcggccgctgtctc
f-pJ/E61A	g*ca*ttcccaacggggacggatatg
r-pJ/E61A	gagcccgtattcggccgctgtc

f-pJ/E69A	g*ca*cgcgtcgagtccgccg
r-pJ/E69A	catatccgtccccgttgggaactcg
f-pJ/A74D	g*a*cgcgaatatctcaccggaccag
r-pJ/A74D	ggactcgacgcgctccatatcc

p-*gvpJ* deletions in pBSK(II)+ and pJAS35

f-Start+PstI-pJxpBSK+	catctgcagcccggg
r-Δ3N-pJ	cccaaaccgacgcgc
r-Δ4N-pJ	aaaccgacgcgctcg
r-Δ5N-pJ	ccgacgcgctcg
r-Δ6N-pJ	acgcgctcgcagg
r-Δ9N-pJ	cagggcgacctcgc
r-Δ12N-pJ	ctcgccgagatgctgg
f-Δ5C-pJ	tgacgtggatgccgtc
f-Δ7C-pJ	ggatgccgtcggcg
f-Δ9C-pJ	cgtcggcgtcgaatcgtc
f-Δ10C-pJ	cggcgtcgaatcgtcc
f-Δ12C-pJ	cgaatcgtccgacagtggg
f-Δ15C-pJ	cgacagtgggttcgtcgac
f-Δ20C-pJ	cgactcggtctccgattgg
f-Δ25C-pJ	ttgggtttccgacgccg
f-Δ30C-pJ	cgggtcggactggtcc
r-Stop+KpnI-pJxpBSK+	tgaggtacccaattcgccct
5'-PstI-pJ	attc **ctgcag** atg agtgaccccaaaccgac
3'-KpnI-pJ	attc **ggtacc** tca tttggtctcctccgctg
5'-PstI-pJΔ3N	attc **ctgcag** atg cccaaaccgacgc
5'-PstI-pJΔ4N	attc **ctgcag** atg aaa ccg acg cgc tcg
5'-PstI-pJΔ5N	attc **ctgcag** atg ccg acg cgc tcg
5'-PstI-pJΔ6N	attc **ctgcag** atg cccaaaccgacgc
5'-PstI-pJΔ9N	attc **ctgcag** atg cagggcgacctc
5'-PstI-pJΔ12N	attc **ctgcag** atg ctcgccgagatgc
3'-KpnI-pJΔ5C	attc **ggtacc** tca tgacgtggatgccgtc
3'-KpnI-pJΔ7C	attc **ggtacc** tca ggatgccgtcggcg
3'-KpnI-pJΔ9C	attc **ggtacc** tca cgtcggcgtcgaatcgtc
3'-KpnI-pJΔ10C	attc **ggtacc** tca cggcgtcgaatcgtc
3'-KpnI-pJΔ12C	attc **ggtacc** tca cgaatcgtccgacagtggg
3'-KpnI-pJΔ15C	attc **ggtacc** tca cgacagtgggttcgtc
3'-KpnI-pJΔ20C	attc **ggtacc** tca cgactcggtctccg
3'-KpnI-pJΔ25C	attc **ggtacc** tca ttgggtttccgacgc
3'-KpnI-pJΔ30C	attc **ggtacc** tca cgggtcggactgg

Split-GFP plasmids *gvpJ* (substitutions, fragments, deletions)

5'-BspHI-pJ	actgac **tcatga** gtgaccccaaaccg
3'-BamHI-pJΔStop	tggatt **ggatcc** c tttggtctcctccgc
5'-BamHI-pJ	aggtcaa **ggatcc** atgagtgaccccaaac
3'-BlpI-pJ_1-56 +Stop	attc **gctcagc** tca ggccgctgtctcgaac
3'-BlpI-pJ_1-56	attc **gctcagc** ga ggccgctgtctcgaac
3'-BamHI-pJ_1-56	attc **ggatcc** c ggccgctgtctcgaac
3'-KpnI-pJ +Stop	attc **ggtacc** tca ggccgctgtctcgaac
5'-NcoI-pJ_47-114	attc **ccatgg** ccgcgattgcttcg
5'-BamHI-pJ_47-114	attc **ggatcc** atg gccgcgattgcttcg
3'-BlpI-pJ	gtaacct **gctcagc** tcatttggtctcctccg

3'-BlpI-pJΔStop	aagtaacct **gctcagc** cctttggtctcctccgc
3'-KpnI-pJ	attc **ggtacc** tcatttggtctcctccgctg
5'-NcoI-pJΔ3N	attc **ccatgg** ca atg cccaaaccgacgc
5'-BspHI-pJΔ4N	attc **tcatga** aaccgacgcgctcg
5'-NcoI-pJΔ5N	attc **ccatgg** ca atg ccgacgcgctcg
5'-BspHI-pJΔ6N	attc **tcatga** cgcgctcgcag
5'-NcoI-pJΔ12N	attc **ccatgg** ca atg ctcgccgagatgc
3'-BamHI-pJΔ5C	attc **ggatcc** c tgacgtggatgccgtc
3'-BamHI-pJΔ7C	attc **ggatcc** c ggatgccgtcggcg
3'-BamHI-pJΔ9C	attc **ggatcc** c cgtcggcgtcgaatcgtc
3'-BamHI-pJΔ10C	attc **ggatcc** c cggcgtcgaatcgtc
3'-BamHI-pJΔ12C	attc **ggatcc** c cgaatcgtcgacagtggg
3'-BamHI-pJΔ15C	attc **ggatcc** c cgacagtgggttcgtc
3'-BamHI-pJΔ20C	attc **ggatcc** c cgactcggtctccg
3'-BamHI-pJΔ25C	attc **ggatcc** c ttgggtttccgacgc
3'-BamHI-pJΔ30C	attc **ggatcc** c cgggtcggactgg

----

*restriction sites are marked in bold; codons for substituted aa are marked in *italics* and are underlined; nucleotides added are separated by blanks

Supplementary Table S2. Effect of mutations in GvpJ on gas vesicle formation

Substitution	position	Vac/GV*	J/L rf value*
L13A	α1	negative	---
L13I	α1	few	---
A14D	α1	negative	---
A14V	α1	negative	---
E15A/D/R	α1	negative	---
E15Q	α1	negative	higher
M16A	α1	negative	---
L17A	α1	negative	---
E18A/M/D/R	α1	negative	---
E18Q	α1	wt	---
M19A	α1	negative	---
M19E	α1	negative	lower
L20A	α1	negative	lower
L20I	α1	negative	---
L21A/I	α1	negative	---
D22A	α1	negative	---
D22E	α1	negative	higher
D22N	α1	negative	---
D22K	α1	spindle/cylinder	--
K23A	loop1	cylinder/ unstable	---
K23D	loop1	negative	---
G24A	loop1	negative	lower
G24S	loop1	negative	lower
V25A	β1	spindle/ unstable	---
V26A	β1	negative	---
V27A	β1	negative	---
A29D	β1	negative	higher
D30A	β1	negative	---
V33A/D	β1	negative	higher
S34A	β1	wt	---
V35A	β1	negative	---
E39A	loop 2	few	---
L41A	β2	negative	---
E44A	β2	negative	---
L45A	β2	spindle	---
**R**46A	β2	cylinder/ unstable	---
**R**46E	β2	negative	---
**A**47D	β2	negative	higher
**A**48D	β2	negative	higher
**I**49A	β2	negative	higher
**A**50D/E	β2	negative	higher
**A**50Y	β2	negative	---
S51A	β2	negative	higher
F52A	α2	negative	higher
E53A	α2	negative	---
T54A/R	α2	negative	---
A55D	α2	negative	---
A56D	α2	negative	---
E57A	α2	negative	---

Y58A	α2	negative	---
Y58E	α2	negative	lower
G59A	α2	negative	---
L60A	α2	negative	higher
E61A	α2	negative	---
E69A	α2	cylinder	---
A74D	loop3	negative	---
J_{WT}		wt	---

*Vac phenotype: wt, wild type; few = few gas vesicles; Rf --- = similar to wild type
GV, gas vesicle shape

Supplementary Table S3. Split-GFP analyses investigating the J_{mut}/L or M_{mut}/L. The fluorescence was measured in LAU/mm^2 and the relative fluorescence was calculated.

transformant		LAU/mm^2	σ LAU/mm^2	Relative fluorescence (rf)
L/J substitution interactions				
$_N$L	J$_C$	311,201	18,493	13.14
	J_L13A$_C$	307,723	16,542	12.98
	J_L13I$_C$	331,351	46,342	12.93
	J_A14D$_C$	290,015	9,074	11.19
	J_A14V$_C$	283,532	7,068	10.92
	J_E15A$_C$	252,666	4,890	9.62
	J_E15D$_C$	278,911	25,654	11.80
	J_E15Q$_C$	372,085	15,175	16.08
	J_E15R$_C$	243,433	26,870	10.17
	J_M16A$_C$	223,804	10,811	9.27
	J_L17A$_C$	365,522	16,107	14.37
	J_E18A$_C$	248,551	46,525	9.45
	J_E18D$_C$	337,776	32,110	14.50
	J_E18M$_C$	287,899	20,087	11.10
	J_E18Q$_C$	215,660	16,396	8.90
	J_E18R$_C$	219,553	12,849	9.08
	J_M19A$_C$	285,595	23,742	11.01
	J_M19E$_C$	192,425	10,140	7.09
	J_L20A$_C$	217,108	8,867	8.13
	J_L20I$_C$	276,275	5,890	13.72
	J_L21A$_C$	157,981	17,245	8.99
	J_L21I$_C$	268,198	37,856	13.29
	J_D22A$_C$	308,235	19,802	11.93
	J_D22E$_C$	315,506	17,616	15.81
	J_D22K$_C$	325,261	19,633	12.64
	J_D22N$_C$	289,244	14,942	14.41
	J_K23A$_C$	349,753	32,151	13.67
	J_K23D$_C$	305,054	32,292	11.80
	J_K23R$_C$	282,251	12,297	14.04
	J_G24A$_C$	179,939	5,505	7.24
	J_G24S$_C$	142,557	4,034	6.60
	J_V25A$_C$	208,952	15,653	11.00
	J_V26A$_C$	311,333	54,863	13.25
	J_V27A$_C$	292,917	31,521	13.21
	J_A29D$_C$	545,660	27,948	25.47
	J_D30A$_C$	198,692	21,742	10.41
	J_V33A$_C$	402,647	49,455	18.53
	J_V33D$_C$	381,461	37,720	17.50
	J_S34A$_C$	339,263	13,976	13.23
	J_V35A$_C$	307,214	18,694	11.89
	J_E39A$_C$	266,255	7,059	9.50
	J_L41A$_C$	369,689	11,923	13.57
	J_E44A$_C$	251,083	22,227	9.63
	J_L45A$_C$	314,214	37,655	12.30
	J_R46A$_C$	342,575	10,300	13.50
	J_R46E$_C$	322,779	23,409	12.66
	J_A47D$_C$	466,101	19,411	21.61
	J_A48D$_C$	353,028	30,056	16.13
	J_I49A$_C$	392,753	27,279	18.05
	J_A50D$_C$	376,845	15,255	17,28
	J_A50E$_C$	353,769	16,645	16.16
	J_A50Y$_C$	255,749	16,234	11.41
	J_S51A$_C$	349,028	17,174	17.42
	J_F52A$_C$	391,868	19,880	19.68
	J_E53A$_C$	307,388	5,887	12.01
	J_T54A$_C$	316,080	16,789	12.38
	J_T54R$_C$	304,508	14,675	11.89
	J_A55D$_C$	233,848	4,217	12.43
	J_A56D$_C$	299,073	8,699	11.66
	J_E57A$_C$	284,417	5,288	11.04
	J_Y58A$_C$	201,304	6,846	10.56
	J_Y58E$_C$	209,186	20,640	7.86
	J_G59A$_C$	295,449	20,047	15.14
	J_L60A$_C$	307,556	6,128	15.80
	J_E61A$_C$	256,080	12,933	12.99
	J_E69A$_C$	275,470	11,256	14.05
	J_A74D$_C$	257,287	31,325	13.05

transformant		LAU/mm^2	σ LAU/mm^2	Relative fluorescence (rf)
L/J deletion interactions				
$_N$L	J$_C$	341,352	9,866	15.72
	J_Δ4N$_C$	482,955	22,051	24.73
	J_Δ6N$_C$	242,331	23,812	12.21
	J_Δ5C$_C$	304,823	17,586	15.62
	J_Δ7C$_C$	315,044	27,465	16.18
	J_Δ9C$_C$	354,435	10,998	18.32
	J_Δ12C$_C$	320,370	9,872	16.47
	J_Δ15C$_C$	262,504	7,931	13.31
	J_Δ20C$_C$	348,166	22,914	17.98
	J_Δ25C$_C$	279,370	8,039	14.23
	J_Δ30C$_C$	268,576	6,970	12.16
L/M substitution interactions				
L$_C$	M$_C$	199,613	34,656	7.77
	M_V15E$_C$	173,052	64,784	6.45
	M_L19E$_C$	177,901	17,921	7.48
	M_G22D$_C$	231,066	27,107	9.58
	M_A23D$_C$	252,635	49,527	9.65
	M_V24D$_C$	290,910	99,595	11.23
	M_R44E$_C$	162,403	22,786	7.54
	M_A45E$_C$	366,492	144,119	17.95
	M_A46E$_C$	155,180	21,823	5.57
	M_I47A$_C$	191,650	6,552	7.16
	M_A48D$_C$	177,806	19,685	7.45
L/M1-25 substitution interactions				
$_N$L	M$_N$	1,682,869	541,810	65.25
	M_E02A$_N$	1,283,829	244,490	67.15
	M_P03A$_N$	1,594,677	50,768	84.10
	M_T04A$_N$	1,414,952	273,203	69.90
	M_K05A$_N$	1,526,740	209,660	77.64
	M_D06A$_N$	1,247,417	111,424	65.57
	M_E07K$_N$	124,587	106,469	80.46
	M_T08A$_N$	2,141,202	159,651	113.26
	M_H09A$_N$	1,220,235	133,869	62.05
	M_A10D$_N$	1,184,781	261,837	58.23
	M_I11A$_N$	1,641,347	189,102	84.36
	M_I11D$_N$	2,073,193	209,138	106.83
	M_V12Y$_N$	1,162,180	174,316	58.13
	M_E13A$_N$	1,154,350	28,032	60.60
	M_F14A$_N$	1,137,529	213,254	56.98
	M_V15A$_N$	2,104,044	441,164	101.78
	M_D16A$_N$	877,442	101,732	45.82
	M_V17A$_N$	1,414,810	122,470	74.50
	M_L18A$_N$	1,427,971	116,759	75.20
	M_L19A$_N$	1,550,256	55,602	81.73
	M_R20A$_N$	1,049,825	112,269	54.11
	M_D21A$_N$	1,262,809	67,600	66.39
	M_G22A$_N$	1,673,417	136,131	85.47
	M_A23D$_N$	1,778,716	36,033	93.92
	M_V24A$_N$	1,423,728	59,154	74.97
	M_I25A$_N$	1,228,149	88,782	64.54

Supplementary Table S4. Split-GFP analyses investigating interactions of A-J-M fragments. The fluorescence was measured in LAU/mm^2 and the relative fluorescence calculated.

transformant		LAU/mm^2	σ LAU/mm^2	Relative fluorescence (rf)
control				
	WR340	17,066	1,687	0.00
GvpJ interactions				
$_{N}$J	A1-22$_{C}$	15,515	1,185	0.06
	$_{C}$A1-22	13,310	1,117	0.00
J$_{N}$	A1-22$_{C}$	13,310	1,117	0.00
	$_{C}$A1-22	12,931	1,974	0.01
$_{C}$J	A1-22$_{N}$	15,603	779	0.05
	$_{N}$A1-22	17,016	637	0.14
J$_{C}$	A1-22$_{N}$	25,287	2,336	0.70
	$_{N}$A1-22	40,430	2,947	1.71
$_{N}$J	A44-76$_{C}$	11,772	569	0.10
	$_{C}$A44-76	10,993	893	0.05
J$_{N}$	A44-76$_{C}$	10,841	867	0.04
	$_{C}$A44-76	11,023	1,328	0.07
$_{C}$J	A44-76$_{N}$	11,040	1,271	0.06
	$_{N}$A44-76	9,293	2,029	0.01
J$_{C}$	A44-76$_{N}$	21,842	1,550	1.03
	$_{N}$A44-76	16,121	1,185	0.50
$_{N}$J	M1-25$_{C}$	16,685	1,969	0.66
	$_{C}$M1-25	11,355	1,839	0.14
J$_{N}$	M1-25$_{C}$	16,946	951	0.68
	$_{C}$M1-25	12,195	1,030	0.21
$_{C}$J	M1-25$_{N}$	11,296	1,514	0.15
	$_{N}$M1-25	10,935	1,523	0.10
J$_{C}$	M1-25$_{N}$	15,022	963	0.49
	$_{N}$M1-25	15,789	1,493	0.57
$_{N}$J	M60-84$_{C}$	16,396	458	0.01
	$_{C}$M60-84	15,530	622	0.00
J$_{N}$	M60-84$_{C}$	17,799	834	0.08
	$_{C}$M60-84	16,423	1,561	0.03
$_{C}$J	M60-84$_{N}$	15,259	887	0.00
	$_{N}$M60-84	17,011	1,595	0.05
J$_{C}$	M60-84$_{N}$	20,220	2,125	0.23
	$_{N}$M60-84	47,920	3,298	1.91
GvpJ fragments with GvpA/GvpM				
$_{N}$J1-56	A$_{C}$	19,055	726	0.00
	$_{C}$A	14,995	1,428	0.00
J1-56$_{N}$	A$_{C}$	15,844	369	0.00
	$_{C}$A	15,260	750	0.00
$_{C}$J1-56	A$_{N}$	15,867	1,088	0.00
	$_{N}$A	12,791	455	0.00
J1-56$_{C}$	A$_{N}$	14,375	1,302	0.00
	$_{N}$A	14,825	463	0.00
$_{N}$J1-56	A1-22$_{C}$	14,821	859	0.00
	$_{C}$A1-22	12,163	192	0.00
J1-56$_{N}$	A1-22$_{C}$	13,865	559	0.00
	$_{C}$A1-22	12,505	252	0.00
$_{C}$J1-56	A1-22$_{N}$	243,810	6,391	9.06
	$_{N}$A1-22	129,406	114,911	4.54
J1-56$_{C}$	A1-22$_{N}$	243,821	9,884	9.06
	$_{N}$A1-22	19,084	1,041	0.00

transformant		LAU/mm^2	σ LAU/mm^2	Relative fluorescence (rf)
$_{N}$J1-56	A44-76$_{C}$	18,063	1,269	0.00
	$_{C}$A44-76	14,600	861	0.00
J1-56$_{N}$	A44-76$_{C}$	15,315	337	0.00
	$_{C}$A44-76	13,922	1,686	0.00
$_{C}$J1-56	A44-76$_{N}$	16,683	1,749	0.00
	$_{N}$A44-76	16,268	432	0.00
J1-56$_{C}$	A44-76$_{N}$	18,383	534	0.00
	$_{N}$A44-76	18,091	508	0.00
$_{N}$J1-56	M$_{C}$[1]	105,270	3,549	0.34
	$_{C}$M[1]	111,793	11,823	0.42
J1-56$_{N}$	M$_{C}$[1]	84,466	7,502	0.08
	$_{C}$M[1]	118,065	18,329	0.35
$_{C}$J1-56	M$_{N}$[1]	111,356	7,800	0.27
	$_{N}$M[1]	119,482	6,313	0.37
J1-56$_{C}$	M$_{N}$[1]	100,725	3,806	0.15
	$_{N}$M[1]	95,150	2,439	0.09
$_{N}$J1-56	M1-25$_{C}$	291,164	4,975	18.05
	$_{C}$M1-25	298,948	10,258	18.56
J1-56$_{N}$	M1-25$_{C}$	276,530	22,476	17.09
	$_{C}$M1-25	275,376	15,054	17.02
$_{C}$J1-56	M1-25$_{N}$	273,638	10,757	16.90
	$_{N}$M1-25	250,688	5,141	15.40
J1-56$_{C}$	M1-25$_{N}$	286,298	7,624	17.73
	$_{N}$M1-25	284,322	11,170	17.60
$_{N}$J1-56	M60-84$_{C}$	306,437	6,388	20.75
	$_{C}$M60-84	280,733	7,194	18.93
J1-56$_{N}$	M60-84$_{C}$	289,429	4,594	19.55
	$_{C}$M60-84	301,170	8,442	20.38
$_{C}$J1-56	M60-84$_{N}$	283,697	6,585	19.14
	$_{N}$M60-84	289,387	7,955	19.54
J1-56$_{C}$	M60-84$_{N}$	309,739	9,921	20.99
	$_{N}$M60-84	281,190	11,376	18.96
$_{N}$J47-114	A$_{C}$	34,030	3,212	0.14
	$_{C}$A	17,794	886	0.00
J47-114$_{N}$	A$_{C}$	112,572	93,078	2.93
	$_{C}$A	18,683	205	0.00
$_{C}$J47-114	A$_{N}$	36,273	19,382	0.69
	$_{N}$A	19,039	709	0.00
J47-114$_{C}$	A$_{N}$	17,438	353	0.00
	$_{N}$A	18,226	942	0.00
$_{N}$J47-114	A1-22$_{C}$	27,128	310	0.35
	$_{C}$A1-22	12,503	669	0.00
J47-114$_{N}$	A1-22$_{C}$	14,619	613	0.00
	$_{C}$A1-22	12,024	1,194	0.00
$_{C}$J47-114	A1-22$_{N}$	154,695	136,769	5.71
	$_{N}$A1-22	20,322	1,074	0.00
J47-114$_{C}$	A1-22$_{N}$	11,812	812	0.00
	$_{N}$A1-22	16,591	2,831	0.02

[1] = LAU value of *Hfx. volcanii* WR340: 87,354

continued

transformant		LAU/mm^2	σ LAU/mm^2	Relative fluorescence (rf)
$_N$J47-114	A44-76$_C$	26,216	1,771	0.31
	$_C$A44-76	11,494	723	0.00
J47-114$_N$	A44-76$_C$	14,541	320	0.00
	$_C$A44-76	12,025	728	0.00
$_C$J47-114	A44-76$_N$	149,838	132,004	5.51
	$_N$A44-76	19,857	305	0.00
J47-114$_C$	A44-76$_N$	12,264	1,280	0.00
	$_N$A44-76	13,501	364	0.00
$_N$J47-114	M$_C$	35,279	3,507	0.70
	$_C$M	14,559	168	0.00
J47-114$_N$	M$_C$	16,775	219	0.00
	$_C$M	13,815	1,401	0.00
$_C$J47-114	M$_N$	12,745	1,005	0.00
	$_N$M	14,043	751	0.00
J47-114$_C$	M$_N$	13,073	767	0.00
	$_N$M	14,768	562	0.00
$_N$J47-114	M1-25$_C$	317,358	4,410	19.76
	$_C$M1-25	325,525	8,722	20.30
J47-114$_N$	M1-25$_C$	273,832	7,043	16.92
	$_C$M1-25	267,785	13,573	18.01
$_C$J47-114	M1-25$_N$	291,753	14,128	18.09
	$_N$M1-25	273,405	8,627	16.89
J47-114$_C$	M1-25$_N$	306,459	7,336	19.05
	$_N$M1-25	291,715	5,997	18.09
$_N$J47-114	M60-84$_C$	303,601	20,553	20.55
	$_C$M60-84	327,115	5,204	16.84
J47-114$_N$	M60-84$_C$	295,661	12,051	19.99
	$_C$M60-84	324,978	14,970	16.72
$_C$J47-114	M60-84$_N$	305,845	4,929	20.71
	$_N$M60-84	298,693	14,978	20.20
J47-114$_C$	M60-84$_N$	289,178	29,570	19.53
	$_N$M60-84	287,088	7,985	19.38

GvpJ47-114 interactions with accessory Gvp

$_N$J47-114	F$_C$	16,562	860	0.00
	$_C$F	14,278	2,020	0.00
J47-114$_N$	F$_C$	13,824	609	0.00
	$_C$F	13,240	510	0.00
$_C$J47-114	F$_N$	13,716	294	0.00
	$_N$F	13,166	442	0.00
J47-114$_C$	F$_N$	16,247	520	0.00
	$_N$F	15,313	716	0.00
$_N$J47-114	G$_C$	331,093	40,997	14.61
	$_C$G	22,518	878	0.06
J47-114$_N$	G$_C$	21,539	3,383	0.08
	$_C$G	13,455	982	0.00
$_C$J47-114	G$_N$	16,476	1,223	0.00
	$_N$G	13,251	408	0.00
J47-114$_C$	G$_N$	14,915	601	0.00
	$_N$G	11,579	538	0.00

transformant		LAU/mm^2	σ LAU/mm^2	Relative fluorescence (rf)
$_N$J47-114	H$_C$	272,516	6,881	12.36
	$_C$H	20,393	775	0.02
J47-114$_N$	H$_C$	29,273	1,266	0.44
	$_C$H	14,794	827	0.00
$_C$J47-114	H$_N$	15,861	844	0.00
	$_N$H	15,230	975	0.00
J47-114$_C$	H$_N$	14,412	850	0.00
	$_N$H	14,023	1,437	0.00
$_N$J47-114	I$_C$	13,982	1,228	0.00
	$_C$I	24,422	457	0.12
J47-114$_N$	I$_C$	15,227	1,739	0.00
	$_C$I	17,491	1,015	0.00
$_C$J47-114	I$_N$	21,414	222	0.00
	$_N$I	22,665	776	0.05
J47-114$_C$	I$_N$	18,030	1,218	0.00
	$_N$I	18,985	420	0.00
$_N$J47-114	J$_C$	80,475	4,808	0.19
	$_C$J	18,586	691	0.00
J47-114$_N$	J$_C$	19,376	877	0.00
	$_C$J	16,051	1,144	0.00
$_C$J47-114	J$_N$	15,770	699	0.00
	$_N$J	15,141	535	0.00
J47-114$_C$	J$_N$	15,060	1,128	0.00
	$_N$J	16,024	1,276	0.00
$_N$J47-114	K$_C$	101,494	3,641	3.67
	$_C$K	20,158	881	0.00
J47-114$_N$	K$_C$	19,932	661	0.00
	$_C$K	16,395	702	0.00
$_C$J47-114	K$_N$	12,912	694	0.00
	$_N$K	15,697	686	0.00
J47-114$_C$	K$_N$	15,915	483	0.00
	$_N$K	16,293	441	0.00
$_N$J47-114	L$_C$	320,314	10,263	9.68
	$_C$L	24,013	565	0.00
J47-114$_N$	L$_C$	46,604	2,951	0.55
	$_C$L	20,888	361	0.00
$_C$J47-114	L$_N$	112,470	92,340	3.87
	$_N$L	24,841	411	0.06
J47-114$_C$	L$_N$	20,225	481	0.00
	$_N$L	20,794	674	0.00

GvpJ1-56 interactions with accessory Gvp

$_N$J1-56	F$_C$[1]	81,078	24,236	0.15
	$_C$F[1]	108,337	4,428	0.33
J1-56$_N$	F$_C$[1]	66,713	21,996	0.05
	$_C$F[1]	76,244	8,684	0.02
$_C$J1-56	F$_N$[1]	99,538	8,033	0.23
	$_N$F[1]	101,754	9,478	0.25
J1-56$_C$	F$_N$[1]	108,050	6,702	0.33
	$_N$F[1]	95,692	16,835	0.19

continued

transformant		LAU/mm²	σ LAU/mm²	Relative fluorescence (rf)	transformant		LAU/mm²	σ LAU/mm²	Relative fluorescence (rf)
$_N$J1-56	G$_C$[1]	116,329	5,191	0.43	$_N$J1-56	J$_C$	21,523	352	0.17
	$_C$G[1]	100,934	8,438	0.24		$_C$J	12,922	1,854	0.00
J1-56$_N$	G$_C$[1]	95,945	2,425	0.18	J1-56$_N$	J$_C$	21,410	1,938	0.17
	$_C$G[1]	81,555	21,073	0.13		$_C$J	14,624	1,355	0.00
$_C$J1-56	G$_N$[1]	110,939	8,529	0.37	$_C$J1-56	J$_N$	18,658	542	0.02
	$_N$G[1]	94,514	6,221	0.17		$_N$J	18,531	1,072	0.03
J1-56$_C$	G$_N$[1]	101,179	3,363	0.25	J1-56$_C$	J$_N$	16,474	629	0.00
	$_N$G[1]	103,467	2,950	0.28		$_N$J	17,288	1,587	0.01
$_N$J1-56	H$_C$[1]	85,957	15,050	0.12	$_N$J1-56	K$_C$[1]	107,987	1,078	0.37
	$_C$H[1]	73,801	4,650	0.00		$_C$K[1]	104,292	3,765	0.32
J1-56$_N$	H$_C$[1]	85,002	4,650	0.05	J1-56$_N$	K$_C$[1]	94,576	12,779	0.20
	$_C$H[1]	69,432	14,878	0.02		$_C$K[1]	83,818	4,811	0.07
$_C$J1-56	H$_N$[1]	109,585	3,404	0.35	$_C$J1-56	K$_N$[1]	115,008	8,435	0.46
	$_N$H[1]	103,801	6,612	0.28		$_N$K[1]	64,917	2,803	0.00
J1-56$_C$	H$_N$[1]	101,667	3,018	0.25	J1-56$_C$	K$_N$[1]	105,270	11,477	0.34
	$_N$H[1]	126,969	18,753	0.57		$_N$K[1]	72,108	29,437	0.14
$_N$J1-56	I$_C$[1]	97,928	7,984	0.21	$_N$J1-56	L$_C$[1]	105,270	3,549	0.34
	$_C$I[1]	97,230	4,010	0.20		$_C$L[1]	111,793	11,823	0.42
J1-56$_N$	I$_C$[1]	79,291	28,865	0.17	J1-56$_N$	L$_C$[1]	84,466	7,502	0.08
	$_C$I[1]	80,563	30,553	0.18		$_C$L[1]	118,065	18,329	0.35
$_C$J1-56	I$_N$[1]	119,173	6,352	0.51	$_C$J1-56	L$_N$[1]	111,356	7,800	0.27
	$_N$I[1]	91,665	7,211	0.16		$_N$L[1]	119,482	6,313	0.37
J1-56$_C$	I$_N$[1]	114,719	5,457	0.46	J1-56$_C$	L$_N$[1]	100,725	3,806	0.15
	$_N$I[1]	106,568	15,370	0.35		$_N$L[1]	95,150	2,439	0.09

Supplementary Table S5. Split-GFP analyses investigating interactions of GvpF and GvpL fragments. The fluorescence was measured in LAU/mm^2 and the relative fluorescence calculated.

transformant		LAU/mm^2	σ (LAU/mm^2)	Relative fluorescence (rf)	transformant		LAU/mm^2	σ (LAU/mm^2)	Relative fluorescence (rf)
GvpL fragments					${}_{N}$L133-281	K_C	100,620	13,627	6.15
						${}_{C}$K	19,790	1,166	0.41
${}_{N}$L1-134	G_C	50,142	3,124	2.63	L133-281$_N$	K_C	64,662	2,487	3.60
	${}_{C}$G	14,096	266	0.02		${}_{C}$K	19,408	1,092	0.38
L1-134$_N$	G_C	16,343	1,129	0.18	${}_{C}$L133-281	K_N	10,856	2,638	0.00
	${}_{C}$G	15,053	1,148	0.09		${}_{N}$K	14,461	886	0.08
${}_{C}$L1-134	G_N	13,412	387	0.00	L133-281$_C$	K_N	18,570	2,518	0.32
	${}_{N}$G	12,496	620	0.00		${}_{N}$K	29,739	571	1.11
L1-134$_C$	G_N	12,312	317	0.00					
	${}_{N}$G	15,944	2,165	0.16					
					${}_{N}$L133-281	M_C	36,895	900	1.62
						${}_{C}$M	10,323	949	0.00
${}_{N}$L1-134	J_C	34,069	573	0.97	L133-281$_N$	M_C	16,662	560	0.18
	${}_{C}$J	16,779	1,566	0.03		${}_{C}$M	10,619	811	0.00
L1-134$_N$	J_C	15,699	1,027	0.00	${}_{C}$L133-281	M_N	14,825	892	0.00
	${}_{C}$J	15,669	653	0.00		${}_{N}$M	10,023	871	0.00
${}_{C}$L1-134	J_N	11,821	1,168	0.00	L133-281$_C$	M_N	14,410	711	0.00
	${}_{N}$J	15,406	2,638	0.14		${}_{N}$M	13,218	2,063	0.00
L1-134$_C$	J_N	10,553	1,233	0.00					
	${}_{N}$J	12,335	825	0.00					
					GvpF fragments				
${}_{N}$L1-134	K_C	53,021	3,032	2.84	${}_{N}$F1-110	A_C	21,277	1,773	0.39
	${}_{C}$K	14,979	405	0.08		${}_{C}$A	17,132	1,592	0.12
L1-134$_N$	K_C	15,034	392	0.09	F1-110$_N$	A_C	20,238	1,677	0.32
	${}_{C}$K	13,236	397	0.00		${}_{C}$A	18,346	1,568	0.20
${}_{C}$L1-134	K_N	12,589	1,053	0.04	${}_{C}$F1-110	A_N	20,836	206	0.36
	${}_{N}$K	13,249	381	0.00		${}_{N}$A	20,129	912	0.32
L1-134$_C$	K_N	14,506	648	0.05	F1-110$_C$	A_N	18,209	1,457	0.19
	${}_{N}$K	13,432	1,679	0.05		${}_{N}$A	20,154	1,399	0.32
${}_{N}$L1-134	M_C	23,506	4,905	0.36	${}_{N}$F1-110	A1-22$_C$	21,403	931	0.40
	${}_{C}$M	12,265	1,518	0.00		${}_{C}$ A1-22	20,632	636	0.35
L1-134$_N$	M_C	12,682	581	0.00	F1-110$_N$	A1-22$_C$	21,243	576	0.39
	${}_{C}$M	11,926	968	0.00		${}_{C}$ A1-22	20,265	2,809	0.32
${}_{C}$L1-134	M_N	11,619	585	0.00	${}_{C}$F1-110	A1-22$_N$	21,316	483	0.39
	${}_{N}$M	11,598	318	0.00		${}_{N}$ A1-22	17,377	1,284	0.14
L1-134$_C$	M_N	11,452	1,077	0.00	F1-110$_C$	A1-22$_N$	20,320	1,597	0.33
	${}_{N}$M	12,740	1,072	0.00		${}_{N}$ A1-22	24,176	831	0.58
${}_{N}$L133-281	G_C	48,561	3,402	1.82	${}_{N}$F109-221	A_C	13,664	1,480	0.06
	${}_{C}$G	13,734	378	0.00		${}_{C}$A	10,712	575	0.00
L133-281$_N$	G_C	27,798	1,177	0.56	F109-221$_N$	A_C	12,824	704	0.01
	${}_{C}$G	14,342	380	0.00		${}_{C}$A	12,280	632	0.00
${}_{C}$L133-281	G_N	11,212	1,064	0.00	${}_{C}$F109-221	A_N	9,574	372	0.00
	${}_{N}$G	12,734	1,213	0.00		${}_{N}$A	10,993	449	0.00
L133-281$_C$	G_N	14,209	325	0.00	F109-221$_C$	A_N	14,080	3,218	0.13
	${}_{N}$G	16,994	2,105	0.04		${}_{N}$A	15,133	2,250	0.15
${}_{N}$L133-281	J_C	30,374	1,789	0.71	${}_{N}$F109-221	A1-22$_C$	17,370	773	0.30
	${}_{C}$J	13,146	735	0.00		${}_{C}$A1-22	9,356	726	0.00
L133-281$_N$	J_C	20,040	1,263	0.13	F109-221$_N$	A1-22$_C$	12,889	244	0.00
	${}_{C}$J	12,166	613	0.00		${}_{C}$A1-22	11,514	471	0.00
${}_{C}$L133-281	J_N	12,221	447	0.00	${}_{C}$F109-221	A1-22$_N$	10,052	1,781	0.00
	${}_{N}$J	12,786	603	0.00		${}_{N}$A1-22	14,719	413	0.10
L133-281$_C$	J_N	14,866	612	0.00	F109-221$_C$	A1-22$_N$	11,203	910	0.00
	${}_{N}$J	13,679	327	0.00		${}_{N}$A1-22	53,953	8,730	3.03
